# Inferring the epidemiological benefit of indoor vector control interventions against malaria from mosquito data

**DOI:** 10.1038/s41467-022-30700-1

**Published:** 2022-07-05

**Authors:** Ellie Sherrard-Smith, Corine Ngufor, Antoine Sanou, Moussa W. Guelbeogo, Raphael N’Guessan, Eldo Elobolobo, Francisco Saute, Kenyssony Varela, Carlos J. Chaccour, Rose Zulliger, Joseph Wagman, Molly L. Robertson, Mark Rowland, Martin J. Donnelly, Samuel Gonahasa, Sarah G. Staedke, Jan Kolaczinski, Thomas S. Churcher

**Affiliations:** 1 Imperial College London, London, UK; 2 Centre de Recherches Entomologiques de Cotonou, Cotonou, Benin; 3 London School of Hygiene and Tropical Medicine, London, UK; 4 Centre National de Recherche et de Formation sur le Paludisme, Ouagadougou, Burkina Faso; 5 Institut Pierre Richet, Bouake, Côte d’Ivoire; 6 Centro de Investigação em Saúde de Manhiça, Manhiça, Mozambique; 7 PMI VectorLink Project, Abt Associates, Maputo, Mozambique; 8 ISGlobal, Barcelona, Spain; 9 US President’s Malaria Initiative, USAID, Washington, DC USA; 10 PATH, Washington, DC USA; 11 Liverpool School of Tropical Medicine, Liverpool, UK; 12 Infectious Diseases Research Collaboration, Kampala, Uganda; 13 World Health Organization, Geneva, Switzerland

**Keywords:** Malaria, Ecological epidemiology, Epidemiology

## Abstract

The cause of malaria transmission has been known for over a century but it is still unclear whether entomological measures are sufficiently reliable to inform policy decisions in human health. Decision-making on the effectiveness of new insecticide-treated nets (ITNs) and the indoor residual spraying of insecticide (IRS) have been based on epidemiological data, typically collected in cluster-randomised control trials. The number of these trials that can be conducted is limited. Here we use a systematic review to highlight that efficacy estimates of the same intervention may vary substantially between trials. Analyses indicate that mosquito data collected in experimental hut trials can be used to parameterize mechanistic models for *Plasmodium falciparum* malaria and reliably predict the epidemiological efficacy of quick-acting, neuro-acting ITNs and IRS. Results suggest that for certain types of ITNs and IRS using this framework instead of clinical endpoints could support policy and expedite the widespread use of novel technologies.

## Abstract (web summary)

Estimating the effectiveness of malaria vector control interventions has typically relied on resource-intensive cluster randomised trials. Here, the authors estimate changes in malaria prevalence using entomological data from experimental hut trials, which may provide an alternative route to approval of interventions in some situations.

## Introduction

New vector control tools are urgently needed to control malaria^1^. Two sets of evidence on the likely impact of new classes of intervention are required to expedite the time between their development and a World Health Organization (WHO) recommendation for their widespread use; (*i*) information on the tools safety, quality and entomological efficacy and (*ii*) evidence that it reduces disease in the target population^2^. Requirement *i* uses evidence of vector control efficacy pertaining to entomological outcomes and formulation durability. Evidence requirement *ii* needs epidemiological data from human populations where the intervention has been used. Cluster-randomised control trials (RCTs) are the primary method used to generate quality evidence of disease control for interventions, which act to reduce transmission across the whole community and not just those people using them. Provided that the two WHO evidence standards are met, the resulting prequalification of the product by WHO provides the confidence sought by countries and large international procurers such as The Global Fund. To provide some reassurance of generalisability of impact requirement *ii* must have data from a minimum of two epidemiological trials conducted in different settings. There are no specific guidelines on how different these settings need to be, and it is not possible to capture the diverse array of ecological, entomological and epidemiological scenarios the interventions are likely to be deployed in. Unless these data are generated concurrently or shortly after requirement *i*, delays between product development and approval can occur, slowing product uptake and public health impact^3^.

Indoor vector control tools that kill mosquitoes and aim to provide population-level impact in addition to personal protection are the most widely used form of global malaria prevention^4^. Insecticide-treated nets (ITNs) are the principal intervention, with over two billion nets distributed globally by 2020. Until that time, nearly all nets deployed have been broadly equivalent in their design, containing a single insecticide of the pyrethroid class. Resistance to pyrethroids is now widespread^4^ and the WHO has identified the development of nets treated with insecticides other than pyrethroids as an unmet public health need and alternatives are currently under development or evaluation. The synergist piperonyl butoxide (PBO) has been added to some pyrethroid net products to combat pyrethroid-resistant mosquitoes since 2008. Pyrethroid-PBO ITNs have only been widely distributed since 2018 following demonstration of their impact on disease^5,6^, which led to a conditional WHO recommendation in 2017. The epidemiological evidence from these RCTs is consistent with entomological data that shows the ability of pyrethroid-only ITNs to kill pyrethroid-resistant mosquitoes has been reduced, and this mortality-inducing effect can be somewhat restored with pyrethroid-PBO ITNs, though other explanations have been proposed^7^. A second key vector control tool recommended for large-scale deployment is indoor residual spraying (IRS) of insecticides aimed at killing mosquitoes resting on treated surfaces. Five chemical classes of insecticide are covered by the WHO recommendation for IRS^8^. These products have different durations of activity at killing and inhibiting blood-feeding of mosquitoes^9^, and use and price varies substantially^10^. Overall, IRS has been deployed to protect fewer people than ITN campaigns^10^, though it has been shown to be highly effective in the focal areas where it is used^11^. Malaria budgets are generally restricted, and new(er) ITN and IRS products tend to cost more, at least during market introduction. Post-deployment epidemiological research and surveillance is not always possible in many malaria endemic regions due to a lack of financial resources. It is unclear whether routine case-reporting is sufficiently robust to guide intervention deployment^12^, making the generation of a strong evidence-base essential before new products are adopted.

Surrogates of protection are widely utilised in medicine^13^. Changes in blood-pressure are used to indicate differing risks of hypertension whilst antibody responses provide evidence of vaccine protection. This raises the question whether entomological data on the ability of a tool to kill mosquitoes can be used to infer protection provided to humans. Clinical surrogates used to evaluate drugs and vaccines only need to consider congruence within individual humans, making it easy to link a person’s disease status to whether or not they have received treatment. Extrapolating the impact of interventions on mosquitoes to changes in the burden of disease in populations of people will likely be more complex. We do not therefore refer to entomological measures as a surrogate but rather as measuring a correlation of protection.

Experimental hut trials (EHTs) are a complex real-world entomological assay that quantifies the host-seeking mosquito interaction with humans and indoor vector control interventions. EHTs are conducted in specially designed huts containing volunteers either protected by the intervention or acting as a control (unprotected or, more commonly, sleeping under an untreated net^14^). Wild, free-flying mosquitoes naturally enter huts and differences in numbers caught, dying, and blood-feeding between intervention and control arms are used to estimate entomological efficacy of ITNs and IRS. EHT are widely used in the development of novel ITNs and IRS and follow a well-defined protocol and analysis plan. A standard set of holes are cut into ITNs to mimic natural wear and tear and enable the actions of the insecticide to be fully assessed. Results can be used to parameterise mechanistic models of malaria transmission that capture the different entomological effects of ITNs and IRS in specified local settings^9,15,16^. In terms of speed and cost, these trials sit between entomological laboratory assays and RCTs. To date, there are no published studies, which conducted EHTs alongside epidemiological trials.

Here, we propose a framework to investigate the utility of entomological data in predicting the epidemiological impact of ITNs and IRS recommended for large-scale deployment against malaria (Supplementary Fig. S1). We use this framework to investigate the ability of data derived from EHTs to predict changes in malaria parasite prevalence measured in RCTs. The non-linear transmission dynamics of malaria means that a reduction in mosquito bites caused by ITNs, or IRS, does not correspond to a similar reduction in malaria burden. To account for this, we use a malaria transmission dynamics model to convert EHT data into estimates of epidemiological impact. The model is calibrated to each trial with local entomological and epidemiological data so that it recreates the observed baseline parasite prevalence estimates. It is then run for the duration of the trial and model predictions are compared to observed changes in malaria parasite prevalence at the respective timepoints that match cross-sectional surveys completed throughout each trial. The effectiveness of ITNs and IRS will likely vary depending on local epidemiology, history of control and local mosquito characteristics. We use a systematic review of the published literature to identify all RCTs investigating the effectiveness of mass use of ITNs and IRS at reducing malaria prevalence. This is used to demonstrate how ITN and IRS efficacy varies between sites and provides a robust method for assessing the ability of the framework to predict the effectiveness of ITN and IRS across the range of African sites where RCTs have been conducted.

## Results

### Differences in the effect sizes of ITN and IRS RCTs

**Fig. 1:**
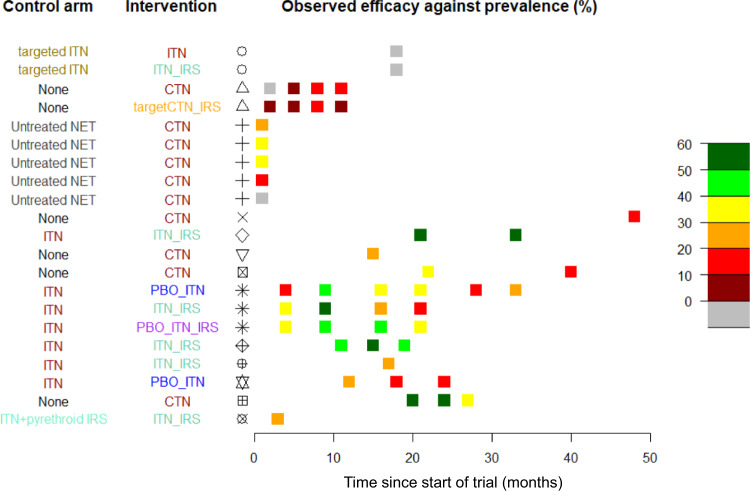
Summary of the randomised control trials completed on ITNs, indoor residual spraying (IRS) or a combination of these intervention tools. The first column indicates the control arm interventions to which the tested intervention (2nd column) are compared. Intervention types represented include no-intervention (black), untreated mosquito nets (grey), conventional nets dipped in pyrethroid insecticide every 6–8 months (CTNs, red), pyrethroid-only insecticide-treated nets, which incorporate insecticide (ITNs, red), pyrethroid-PBO ITNs (blue), or ITNs together with IRS (pyrethroid-only ITN + IRS, pale green, pyrethroid-PBO ITN + IRS, purple) or IRS only (orange). The country and study represented are shown in columns 3 and 4; symbols correspond to the studies shown in Fig. 2 and references in the supporting information Supplementary Table 1. The efficacy estimate reported in each of the trials is shown by the coloured square box at the appropriate timepoint the survey was conducted following start of the trial. It is calculated as the mean difference between reported malaria prevalence in the intervention arm relative to the control arm, with greener colours indicating higher observed differences. Trials vary substantially in the number and timing of the cross-sectional surveys.

**Fig. 2:**
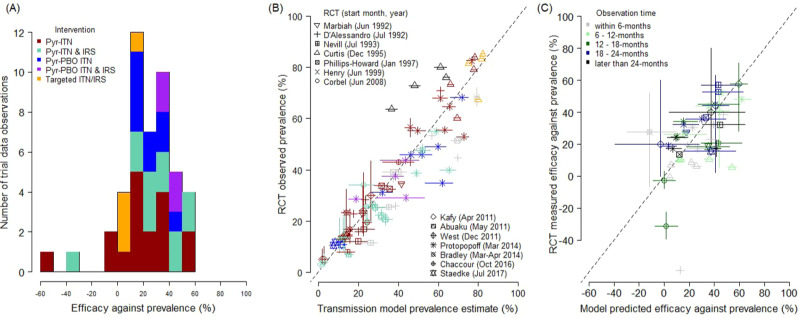
Differences in the epidemiological impact of insecticide-treated nets (ITNs) and the residual spraying of insecticides indoors (IRS) as evaluated in cluster-randomised control trials (RCTs) and predicted by a entomological data. **A** Trial observed relative (to respective control arms as noted in Fig. 1, and Materials and Methods) efficacy against prevalence estimated for 46 data observations (Supplementary Data S1.8). Bar colours indicate the different types of intervention examined. **B** Comparison between observed trial prevalence and prevalence predicted by the transmission dynamics model parameterised using entomological data (matching diagnostic method and cohort characteristics, Supplementary Data S1.7, best-fitting parameters shown; Supplementary Table 3, column 4) for 13 RCTs, with symbols identifying principal investigators listed with the start date of the trial, that reported a total of 73 prevalence cross-sectional surveys. Colours indicate the type of intervention in the trial arm: pyrethroid-only nets (red), pyrethroid-PBO nets (blue), pyrethroid-only nets and IRS (green), pyrethroid-PBO nets and IRS (purple), or IRS only (orange). **C** Comparison of observed efficacy estimates and those predicted by the model (Supplement Data S8). In **C**, colours denote the length of time in months since the deployment of interventions when the prevalence observation was made that was used to estimate efficacy. Individual model predictions for each study are given in Supplementary Figs. S3–S15 with equivalent figures for alternative methods of combing data shown in Supplementary Fig. S18. Vertical and horizontal solid lines around point estimates (mean) for either observed or predicted data indicate 95% uncertainty from intervention performance, while dashed black line in **B** and **C** show the equivalence line. Uncertainty estimates for the observed data found in Supplementary Data S1.3 and for the different models in Supplementary Data S1.7 and 1.8 for **B** and **C**, respectively.

Data from a limited number of epidemiological trials cannot be readily extrapolated to infer the quantitative impact of ITNs and IRS in different settings. Thirteen different RCTs (Supplementary Table 1) were identified by the systematic review that fulfil the search criteria and recorded changes in malaria parasite prevalence following the mass use of ITNs and/or IRS (Supplementary Fig. S2). These studies contained 37 distinct trial arms (a total of 73 cross-sectional surveys) implementing different ITN and IRS products alone, or in combination, across multiple ecological settings in Africa (Supplementary Data 1.1–1.3). The ability of interventions to reduce malaria prevalence relative to the control arm varied markedly across RCTs (Fig. 1). Differences in trial design and study setting were considerable, with the time of impact assessment after deployment of the interventions varying substantially, making overall comparison difficult. We do not have cluster-level data to adjust for known ecological differences in trial arms so the effect sizes of the RCTs are crudely estimated as the absolute difference in prevalence of the treatment arm relative to the control arm. Even when consistent endpoints were used, considerable differences between trials remain; for example, in locations with no evidence of insecticide resistance the efficacy of a single brand of net in reducing disease prevalence varied from 11% after 11 months in Tanzania (latest observation reported in the RCT)^17^ to 57% after 20 months in Kenya (earliest observation reported in the RCT)^18^. Multiple entomological and epidemiological determinants may explain these differences, and as noted, with cluster-level data some of these can be accounted for, but the ambiguity of the intervention efficacy (Fig. 2a) will likely hinder extrapolation of the findings to other settings making it more challenging for decision makers to decide on the most appropriate vector control to implement.

### Predicting epidemiological outcomes from entomological data

We systematically investigated the ability of EHT data and models to predict epidemiological outcomes.Each of the 37 trial arms were simulated separately using observed patterns of intervention use (Supplementary Figs. S3–S15). Model predictions were compared to observed trial results for subsequent timepoints. The best-performing model (Supplementary Tables 2 and 3) predicted malaria prevalence at different timepoints after the start of the trials with high accuracy (Fig. 2b, Adjusted-*R*^2^ = 0.95, *N* = 73). Model predictions of epidemiological impact relative to the control arm of the matched cross-sectional survey were broadly consistent with those observed in the RCTs (Fig. 2c, Adjusted-*R*^2^ = 0.67). Further investigation of the goodness-of-fit of each of the different studies investigating the impact of different EHT design (Supplementary Fig. S15) are provided in Supplementary Figs. S17–19. The framework predicted different types of ITNs and IRS vector control interventions with broadly equivalent consistency (Supplementary Table 3), be it the change in malaria parasite prevalence caused by any net (including conventional dip-nets; *R*^2^ = 0.97, *n* = 37), pyrethroid-only long-lasting insecticidal nets (*R*^2^ = 0.97, *n* = 14), pyrethroid-PBO ITNs (*R*^2^ = 0.93, *n* = 7), or additional protection from IRS (*R*^2^ = 0.91, *n* = 20). This analysis suggests that EHTs are equally good at predicting trials of all ITNs and IRS currently recommended for large-scale deployment (though impact following routine deployment is likely to be different).

The rationale for having different experimental hut trial designs is that housing type varies between regions and that this could influence the entomological impact of ITNs and IRS. The analyses are repeated to investigate whether models characterising the entomological impact of ITNs and IRS using EHTs of the regional design are better able to predict RCTs from that region (i.e., do East African design huts better predict the RCTs carried out in East Africa?). Though the number of studies are limited there is no systematic evidence to suggest that models fit to data using the local design of hut predict the local RCTs better using this mechanistic framework (Supplementary Table 4).

## Discussion

There is a need to quickly evaluate the likely epidemiological impact of new ITNs and insecticides for IRS to inform policy. The systematic review of RCT data shows that mass use of ITNs and IRS consistently reduce malaria parasite prevalence, but the magnitude of the decrease varies substantially (Fig.1). This provides evidence that these interventions have public health benefit but that the level of protection can vary due to varying ecologies and endemicities in the setting. RCTs cannot be conducted across the range of areas within which ITNs and IRS could be beneficial, so entomological assays and modelling could be a feasible alternative to help guide local decisions. A previous review^19^ indicated that since 1988, over 136 EHTs of ITN or IRS products have been conducted using broadly standard methodology in over 33 sites in Africa (Supplementary Fig. S14), compared to the 14 RCTs of the same products (since 1992, from 13 sites across the continent of Africa, Supplementary Table 1). This study shows that a framework that combines meta-analyses of EHT data with a transmission dynamics mathematical model can approximate the results of the RCTs for the different ITN and IRS interventions currently widely used.

The trials summarised in this systematic review were conducted over a 30-year period and differed substantially in their design and time of data collection. Earlier trials may not have adhered to currently expected standards as future RCTs must now be registered, and their design reviewed by the WHO Vector Control Advisory Group and other bodies in advance to ensure they are robust. Despite this, the substantial uncertainty in epidemiological effect size outlined in Fig. 1 is generally predictable by the model, which accounts for ecological site-specific details. Meta-analyses can deal with differences by sub-group analyses though this is heavily restricted when the number of trials is small^20^. Given that WHO’s minimum requirement for evaluation of a new intervention is only two trials with epidemiological outcomes, it is notable that observed differences between trials can be largely explained using EHT data and trial context. Nevertheless, the question arises whether EHTs are robust enough to support a policy decision? Considering the extensive entomological evidence-base for the ITNs and IRS and the wide variability observed in RCT efficacy estimates our analysis suggests that, for those interventions examined here (fast-acting and neuro-acting insecticide-treated nets and spray products), the evidence is sufficiently strong to justify using entomological efficacy measured in EHTs as a correlate of protection to facilitate WHO recommendation on whether a product in an existing product class would have epidemiological value (evidence requirement *ii*). It is important to highlight that the interventions investigated here all have proven epidemiological impact, so the ability of EHTs to identify interventions that do not provide epidemiological benefit (should that be shown from epidemiological data) has not been tested. Such an acceptance of entomological data would bring ITNs in line with IRS evaluation as new IRS products with proven quick-acting entomological characteristics do not require epidemiological evidence of impact. Generation and use of high-quality information on the epidemiological impact of vector control interventions should always be encouraged to support decision-making. This work suggests that in the absence of these data EHT results combined with local information can predict the magnitude of epidemiological impact. It also justifies the use of EHT entomological data to evaluate the non-inferiority of new products that are like those that have already provided epidemiological evidence of impact^21^.

There are several important caveats to the use of EHT data to support decision-making. The evidence presented here is for ITN and IRS products with very defined entomological modes of action that use quick-acting neuro-acting insecticides to kill and inhibit blood-feeding—effects that are measurable using experimental huts. Outcomes which are not captured by experimental huts may fail to identify epidemiological impact. ITNs with different modes of action, such as the pyrrole insecticides^22^ that act on mitochondrial respiratory pathways or insect growth regulators^23^, which act on female mosquito fertility will require further empirical epidemiological evaluation to allow analysis similar to the one presented here. Similarly, vector control tools other than ITNs and IRS with alternative delivery mechanisms, like spatial repellents or attractive targeted sugar baits, will require extensive epidemiological evidence to support their use (ideally using RCTs). The use of entomological assays to evaluate these new types of interventions alongside their epidemiological trials (in the same trial sites) could provide the evidence-base to support using mosquito data as a correlate of protection for evaluating novel methods of vector control in the future. Here, we have only considered African ITN and IRS trials, and additional studies are needed to underpin potential extrapolation of impact to other malaria endemic parts of the world. The model is specific to falciparum malaria, so we cannot comment on the use of this methodology for other *Plasmodium* parasites of public health importance. Results indicate that using a hut design from the region where the RCT took place (East or West Africa) did not improve model predictions. No design is going to broadly capture the diversity of housing from such a large and varied geographical continent and the diversity of mosquito species within that region could dramatically impact ITN and IRS efficacy. Studies directly comparing hut designs would be interesting to explore the advantages of tailoring the assay to the local regional housing compared to having a more consistent assay between sites, which might allow a more direct comparison of the same product against different mosquito populations. Further work is also needed to verify the durability of pyrethroid-PBO ITNs and assess whether the natural aging process can be artificially induced by washing (as is the case for pyrethroid-only ITNs). An artificial method of aging ITNs would enable new and washed nets to be simultaneously evaluated in EHTs allowing nets to be evaluated over a couple of months rather than multiple years in RCTs.

If EHT data is to be increasingly used to support policy, then there is a further need to ensure reproducibility of results. The WHO already require EHTs used in vector control product registration to follow good laboratory practice regulations, and there are on-going projects to certify testing facilities for hut sites across Africa. Protocols already provide clear instructions as to how study arms should be selected, rotated, randomised, how study arms can be blinded, and replicated^14^, though power calculations are rarely conducted, primarily due to uncertainties in the numbers of mosquitoes caught per night. This study has tried to reduce any potential bias by using a meta-analysis of many trials. In future the measurement error of the assay needs to be further assessed and causes of variability in trial outcomes identified to instil greater confidence in results from individual trials. This would allow more rigorous power calculations to be conducted, though adaptive trial design may be required to ensure conclusions are based on sufficient numbers of mosquitoes. In this study, EHTs were used for assessing the entomological correlate of protection. There is considerable scope to improve predictions; future studies could consider augmenting EHT data with other laboratory or field assays that can evaluate interventions^24^. These could be rigorously assessed using the framework outlined here.

We stress that epidemiological trials should still be advocated for to evaluate WHO recommended ITN and IRS products. Mosquito ecology is highly diverse, and we do not fully understand how the effectiveness of these interventions vary between settings nor how they are influenced by changing mosquito populations (for example due to insecticide resistance or behavioural avoidance^25^). Further epidemiological studies, such as well-resourced implementation programmes^11^, will be important to verify the context-specific impact estimates needed for intervention prioritisation, and to provide continued justification for the considerable annual cost of vector control. Alongside this, the use of entomological data can expedite the time between the development of new ITNs and IRS and their widespread use, saving lives.

## Methods

### Systematic review

A systematic review (PROSPERO Registered: CRD42020165355) of all cluster-randomised control trials currently published on ITNs [including conventional nets (CTNs), pyrethroid-only long-lasting nets (pyrethroid-nets), and pyrethroid-piperonyl butoxide synergist nets (pyrethroid-PBO ITNs)], IRS or a combination of both interventions was completed to validate an established transmission model for *Plasmodium falciparum* malaria parameterised using entomological assessment of the interventions. Three search platforms, Web of Knowledge, PubMed and Google Scholar were used and further studies were included from three recent Cochrane reviews that have focused on individual- or cluster- randomised control trials testing either ITNs, IRS or both^26–28^. Our search criteria focused on studies within Africa, and those reporting an epidemiological outcome such as parasite prevalence or clinical incidence in a defined age-cohort. A total of 138 studies were initially identified for further assessment (Supplementary Fig. S2).

Those papers identified through the systematic review went through another round of screening to ensure they fell within the scope of the work and were compatible with existing modelling parameterisation. These criteria included (*i*) the intervention falls within an existing World Health Organization recommendation (so trials, or arms of trials, investigating pyrethroid-pyriproxyfen ITNs^29^ or insecticide-treated curtains^30^ were excluded), (*ii*) the entomological impact of the product had been previously statistically characterised as part of the modelling framework (trials investigating DDT^31^ or propoxur IRS^32^ were excluded), (*iii*) the study was within the Africa continent, (*iv*) the study randomised interventions in the intervention arm across the community (i.e., interventions were not targeted to individuals or risk groups within the community)^33–35^, and (*v)* the study was not reporting a cluster-randomised design^36^. A full description of why studies and arms were excluded is provided in Data S1.1.

RCTs can assess the public health impact of interventions using different epidemiological endpoints. The two most common metrics used in malaria RCTs is infection prevalence (generally assessing parasitemia in a particular age group using microscopy or rapid diagnostic tests) or clinical incidence (typically assessed using active case detection in a cohort, which had previously been cleared of infection). These metrics are both equally valid though may give different results. For example, it may be harder to change malaria parasite prevalence with a partially effective intervention in a high-transmission setting (where people have a high chance of being reinfected) compared to a low-transmission setting (where reinfection is less common). Similarly, estimates of clinical incidence will vary depending on the study design and regularity of follow-up. For example, there are practical constraints on the number of times people within an active cohort can be tested. In areas of higher transmission incidence estimates will be greater the more regularly the cohort is tested as people infected multiple times between screening will be less common. This information on the regularity of screening is not always reported making it difficult to adjust models accordingly. It is also important to account for cluster-level effects when interpreting trial results, and this cluster-level data is also mostly unavailable^37^. The systematic review identified more studies that evaluated interventions in their ability to change malaria prevalence, with 13 out of 14 RCTs showing how the intervention changed parasite prevalence between the study arms compared with 8 RCTs, which reported changes in clinical incidence. Therefore, we focus on prevalence as our metric for epidemiology impact in this framework though note this should be repeated with clinical incidence estimates should more data become available. The final dataset had 73 cross-sectional surveys of prevalence in a defined age-cohort, 37 trial arms from 13 different RCTs.

### Characterising the entomological impact of ITNs and IRS

Experimental hut trials (EHTs) measure the outcome of wild, free-flying, mosquito attempting to feed on volunteers resting indoors in the presence of an indoor intervention^38^. This includes (*i*) whether or not a mosquito is deterred away from a hut, which has the intervention (calculated by the number of mosquitoes found in the control hut relative to the intervention hut), (*ii*) whether the mosquito exits without feeding (repellence, measured as the percentage of alive unfed mosquitoes inside the intervention hut), (*iii*) the percentage entering the hut that successfully blood-feed, or (*iv*) the percentage of mosquitoes which die. Intervention efficacy is typically summarised for the intervention huts relative to a no-intervention (or untreated net) control huts, be it induced mortality (the increase in the percentage of mosquitoes dying over a 24-h period) or blood-feeding inhibition (the reduction in the percentage of mosquitoes receiving a blood-meal).

EHTs use specially built structures that follow a defined floor-plan and set of specifications. There are multiple designs of experimental hut as they were originally intended to replicate the predominant type of housing found in the local area. We recently conducted a systematic review to capture the average behaviours of mosquitoes across different hut designs^19^. The two most used huts in Africa are the West African design and East Africa hut^39^ (a third hut—the Ifakara hut—is not considered here^39^). The meta-analyses showed that the associations describing the probable outcome of a mosquito feeding attempt (deterrence, repellence, successful feeding, or death) varies according to hut design. It is unclear that hut design best predicts epidemiological impact.

Meta-analyses of EHT data have shown how the entomological efficacy of pyrethroid-nets has diminished over time, probably due to the rise of pyrethroid-resistant mosquitoes^16,19,40^, though there may be some manufacturing changes^41^. EHTs are conducted throughout Africa but are limited to the sites where the huts are built and cannot directly inform estimates of ITN efficacy outside of these areas. The most widely used quantitative measure for approximating the phenotypic level of resistance in the local mosquito population is the discriminating-dose bioassay. There are two main types of discriminating assays, the WHO susceptibility bioassay and the CDC bottle bioassay^42,43^. Both these assays measure the proportion of local *Anopheline* mosquitoes that survive 24-h following exposure to a discriminatory dose of pyrethroid for 60 min. Results from these bioassays are highly variable^44^ though collating data from multiple tests has shown clear trends over time^45^. The relationship between the level of resistance in the local mosquito population (as measured in a discriminating-dose bioassay) and the mortality induced by ITNs in EHTs can be used to extrapolate the results from hut trials to other geographical regions^16^.

### Modelling rationale

The two main metrics recorded in EHTs do not capture all entomological impacts of ITNs and IRS. Though useful, induced mortality does not consider the sub-lethal impact of interventions whilst blood-feeding inhibition fails to differentiate between preventing blood-meals and killing mosquitoes, which are likely to have very different epidemiological impacts. Killing mosquitoes reduces the force of infection for users and non-users (through a community effect) so the overall effectiveness of treated nets and IRS will vary according to how abundantly and regularly they are used by the local human population. In addition, the impact of ITNs and IRS is likely to vary between sites because of factors such as the disease endemicity itself driven by societal behaviours, seasonality of transmission and the use of other malaria control interventions, amongst others. This means that raw EHT data is unlikely to directly correlate with the results of RCTs.

EHTs are widely used to parameterise malaria transmission dynamics mathematical models^46–48^. These models rigorously quantify the outcome of each mosquito feeding attempt and, by making a limited number of assumptions, can estimate an overall entomological efficacy by combining the impact of the level of personal protection elicited by the intervention to the user and the indirect community effect provided to both users and non-users. Transmission dynamics mathematical models are designed to mechanistically capture the underlying processes governing malaria transmission and so can account for known non-linear processes such as the acquisition of human immunity^49–51^. This enables these models to translate the entomological efficacy quantified in an EHT into predictions of epidemiological impact given the characteristics of the site. Unfortunately, to date, there are no published EHTs that have been conducted alongside RCT evaluation of ITNs or IRS products (and therefore evaluated against the same mosquito population). To overcome this issue we parameterise the models using a meta-analyses of 136 EHT results^16,19^ collated from across Africa, which quantifies how mosquito deterrence, repellence, successful feeding, or death varies with time since the intervention is deployed and according to the level of pyrethroid resistance in the local mosquito population (as measured by the discriminating-dose bioassay). This approach has been able to recreate the epidemiological impact observed in RCTs evaluating a small number of ITNs^15^ or IRS products^9^, but this is the first attempt at using this method to validate the modelling framework against all trials evaluating nets and IRS.

There is considerable uncertainty in how the entomological efficacy of treated ITNs varies with the level of resistance in the local population. This is a key relationship determining how field discriminating-dose bioassay data should be interpreted yet it is highly uncertain, with a recent meta-analyses indicating that it is equally well explained by two different functional forms (the logistic or log-logistic functions)^19^. Similarly, it is unclear whether the epidemiological impact of ITNs or IRS is best captured by all experimental hut data combined (Supplementary Fig. S14C, D)^19^ or if the meta-analyses should be restricted to just West or East African hut design data alone. To rigorously differentiate between these options six different models are run for each trial arm (*n* = 37), varying both the relationship between discriminating-dose bioassay and EHT mosquito mortality (either the logistic or log-logistic function) and the data used in the EHT meta-analyses (all data, East or West African design huts). The ability of these models to recreate the observed results is statistically compared and the most accurate selected for the main analyses.

### Transmission dynamics model

The malaria transmission model that we use here incorporates the transmission dynamics of *Plasmodium falciparum* between human hosts and *Anopheles* mosquito vectors. The differential equations and associated assumptions of the original transmission model^52^ have been comprehensively reported in the Supplementary Material from Griffin et al.^53^, Walker et al.^54^ and Winskill et al.^55^. The model has been extensively fitted to data on the relationship between vector density, entomological inoculation rate, parasite prevalence, uncomplicated malaria, severe disease and death^49,52,53,56,57^. Model equations and assumptions are provided in the Supplementary Methods and https://github.com/jamiegriffin/Malaria_simulation. Unless stated (Supplementary Data S1), default parameters are taken from these papers.

### Data requirements for model simulation

The transmission model can be parameterised to describe the specific ecology of each RCT location using data on the mosquito bionomics, seasonal transmission patterns, historic use of various interventions—principally insecticide-treated ITNs or the residual spraying of insecticides (IRS)—and baseline endemicity. These data are recorded within the research articles reporting the trials at the trial arm level (Supplementary Data S1.2 notes where data are available and which resources were used; Supplementary Data S1.3 lists the key data identified for model parameterisation) and Supplementary Fig. S1 provides a diagram of how they are combined to inform the model.

Briefly, the *Anopheles* mosquito species composition at baseline is used to determine the proportion of mosquitoes with bespoke behaviours that could alter exposure risk to mosquito bites and thus transmission risk. Species-specific mosquito behaviours are parameterised from systematic reviews on anthropophagy, using the human blood index^47,58,59^, and the proportion of mosquito bites that are received indoors or in bed because this impacts the efficacy estimate for indoor interventions^60^.

Other information that are specific to each trial also help interpret our success at predicting, or not, the observed results of an intervention tested in an RCT; the diagnostic used to measure prevalence or incidence is useful because different tests have different sensitivities^61^, which can be included in the model framework^54^. The baseline burden of infection is particularly important to enable the model to be calibrated to the endemicity of the study site by varying the number of mosquitoes per person (the human:mosquito ratio). This is determined by a cross-sectional estimate of parasite prevalence in a defined age-cohort at a particular time of year of the baseline survey.

For any location, the current level of endemicity is determined by the historic interventions already operating at the site. Therefore, wherever possible, ITN use and the historic use of sprayed insecticides, as well as the estimated proportion of clinical cases that are drug-treated, are included as baseline parameters.

In addition to the waning potency of insecticide active ingredient outlined above, the impact of nets can also wane because of changes in the proportion of people using them. This can be driven by the quality of the product, seasonal patterns in humidity or other social patterns of use^62–64^. Where data are available, this waning adherence to net use is captured by fitting an exponential decay function to the proportion of people using nets measured at cross-sectional surveys throughout the trials:

1 $${{{{{{{\mathrm{U}}}}}}{{{{{\mathrm{sage}}}}}}}}_{i}={e}^{-{\sigma }_{i}t}$$

where *σ* is a parameter determining how rapidly people stop using nets in an intervention arm *i* of the trial and *t* is time in years. Parameter estimates for pyrethroid-only and pyrethroid-PBO ITNs are provided for different levels of resistance for the 6 potential methods of associating bioassays and using data (Supplementary Data S1.4).

The IRS product used is equally important as the entomological impact of different products vary, particularly for pyrethroid-based IRS in the presence of resistant mosquitoes^9^. Supplementary Data S1.5 show the parameter estimates for products included in the analysis.

The seasonality of transmission has been defined previously for each RCT site (at the administration subunit 1 level) using normalised rainfall patterns obtained from the US Climate Prediction Center^65^. The daily time series are aggregated to 64 points per year for years 2002 to 2009. A Fourier function is fitted to these data to capture seasonality by reconstructing annual rainfall patterns^54,66^. We deliberately do not match rainfall data from the respective RCTs, which would likely improve the model estimates because we are ultimately testing whether this framework has predictive power across future years or alternative ecologies, where we will not know how rainfall will exactly impact mosquito densities and hence malaria transmission.

### Statistical analysis

The mean simulated malaria prevalence (matching the age-cohort of the trial) is recorded for all RCT surveys timepoints. This equates to a total of 73 cross-sectional surveys post-implementation. The process was repeated using the 6 different entomological parameter sets (the relationship between bioassay and hut trial mortality and the hut design used to summarise treated net entomological impact). An illustration of the different models and their fit to data is demonstrated in Supplementary Fig. S17 for a recent study trialling pyrethroid-only nets, pyrethroid-PBO ITNs alone or in combination with a long-lasting IRS product in Tanzania^5^. The difference between the observed and predicted prevalence at each timepoint is shown for all RCTs in Supplementary Fig. S18. A simple linear regression is conducted comparing observed and predicted results are summarised in Supplementary Table 3. Let *X*_*i*_ denote the malaria prevalence predicted by the model at timepoint *i* while *Y*_*i*_ is the observed prevalence. The regression,

2 $${Y}_{i}=m{X}_{i}$$

for *i* = *1,…,c* + *n*, where *m* is the gradient between the observed and predicted result (consistent across studies), *c* is the number of post-intervention datapoints in the control arms and *n* is the number of post-intervention datapoints in the intervention arms (*c* + *n* = 73 for analyses of all RCTs). Better fitting models have a higher adjusted *R*^2^ (adjusted *R*^2^ values of one indicate the model is perfectly predicting the trial result) whilst the gradient of the regression *m* indicates any bias (with value of one reporting the model can predict prevalence equally well across the endemicity range). Results are presented for all ITNs and IRS RCTs and separately for RCTs of different types of (pyrethroid-only ITNs, pyrethroid-PBO ITNs and IRS, Supplementary Table 3). The log-logistic model (results 4–6 in Supplementary Table 3) describing the relationship between bioassay and hut trial mortality consistently fits the data better, with models fit using either all hut trial data or East African design huts having a similar accuracy (adjusted *R*^2^ = 0.95). This parameter combination also had the least bias, with the best fit regression line being closer to one.

The average efficacy of the different ITNs and IRS combinations was calculated by comparing malaria prevalence for the different trial arms to the respective control arms at matched timepoints following the introduction of interventions. Let ${E}_{{jk}}^{l}$ be the relative reduction in the malaria prevalence between the control (*k* = 0) to intervention (*k* = 1) arms at matched timepoint *j* in the same trial for either the predicted (*l* = *X*_*jk*_*)* or observed (*l* = *Y*_*jk*_*)* malaria prevalence,

3 $${E}_{j}^{X}=({{X}_{j0}-{X}}_{j1})/{X}_{j0}\,{{{{{\rm{ and }}}}}}\,{E}_{j}^{Y}=({Y}_{j0}-{Y}_{j1})/{Y}_{j0}$$

for *j* = *1,…,n*. The goodness of fit for the efficacy estimates is calculated in a similar manner to the prevalence estimates by substituting in ${E}_{j}^{X}$ and ${E}_{j}^{Y}$ into *X*_*i*_ and *Y*_*i*_ in E2, respectively. Models are on average able to estimate the efficacy of the interventions at different timepoints (Supplementary Table 3). Estimates for some timepoints diverge substantially (for example, the study testing conventional nets in the Gambia relative to untreated nets^67^ measured negative effect in one setting; the treated net arm having more infected children whereas the model predicted a 12.5% reduction due to the CTN (with parameters derived from all EHT data and the log-logistic function, 4 in Supplementary Table 3), Supplementary Data S1.8), but in most studies the trial average (averaged across all timepoints) is remarkably consistent. Accuracy is lower than estimates of absolute prevalence, in part because the difference between the percentage of people slide positive in low-endemicity settings may be relatively modest in absolute terms but might represent a substantial difference as a percentage. It is also important to note that when the models do systematically miss some timepoints, this is consistent across the control and treated arms. For example, in the Protopopoff et al. study in Tanzania^5^ (Figs. S14 and S17) efficacy is over-estimated in all arms 18 months after the start of the trial, but the relative difference between the arms (in terms of ordering, and the efficacy estimate) is relatively consistent. This indicates that unmeasured factors, such as differences in the timing and duration of the rainy season, may have occurred across all trial arms. As previously, the log-logistic functional form describing the relationship between bioassay and hut trial mortality consistently fits the data better (Supplementary Table 3, options 4 to 6). The models fit describing the entomological efficacy of any net using all EHT data predicts efficacy data better with East African design hut data providing similar accuracy (adjusted *R*^2^ = 0.64 vs. 0.62, respectively). Following this we select the log-logistic functional form to describe the relationship between mortality in the discriminating-dose bioassay and EHT and characterise the entomological efficacy of treated ITNs using data from both East and West African design huts for the main analyses (Fig. 2B, C).

The ability of the best-performing model (Supplementary Table 3, column 4: log-logistic function and all EHT data) to capture the relative drop in prevalence over time compared to the baseline (pre-intervention) estimate is shown in Supplementary Fig. S19. This value is denoted as ${\dot{E}}_{t}^{l}$ and is calculated as,

4 $${\dot{E}}_{t}^{X}=({X}_{0}-{X}_{t})\,{{{{{\rm{and}}}}}}\,{\dot{E}}_{t}^{Y}=({X}_{0}-{Y}_{t})$$

where ${X}_{0}$ is the malaria prevalence at baseline (prior to intervention deployment with the exception of Chaccour et al.^68^) observed from the RCT and the model is calibrated to this endemicity. *X*_*t*_ is then the subsequent cross-sectional survey observed for each study, and RCTs have different numbers of surveys ranging from 1 to 4 in the published literature. The corresponding model estimate is represented by *Y*_*t*_. Estimates are calculated for all post-intervention timepoints in both control and intervention arms and are shown in Fig. S19A. The difference between ${\dot{E}}_{t}^{X}$ and ${\dot{E}}_{t}^{Y}$ can be used to explore how closely the model is able to predict this absolute difference observed in the trials (a value of 0 indicates exact match, high predictive ability). The model overestimates the performance of IRS only, deployed in 1995 using the pyrethroid IRS ICON CS 10% (Syngenta), but otherwise there is no difference in the models’ ability to estimate different ITN interventions or combination net and IRS interventions, be it the absence of an intervention, conventional dipped-nets, pyrethroid-only nets, pyrethroid-PBO ITNs with or without IRS (Fig. S19B). All code is available^69^.

### Reporting summary

Further information on research design is available in the Nature Research Reporting Summary linked to this article.

## Supplementary information

Supplementary Information

Description of Additional Supplementary Files

Supplementary Data 1

Peer Review File

Reporting Summary

## Data Availability

Results from the systematic review and all data used in the analyses are provided in Supplementary Data; these are collated data from previously published trials that are owned by the authors noted in the publications documented in Supplementary Data.

## Code availability

Model code can be found here: https://github.com/jamiegriffin/Malaria_simulation and data manipulation, input parameters and processing is available here: https://github.com/EllieSherrardSmith/ibm_rct_prediction^69^.
