# Supplementary material for: Inferring the epidemiological benefit of indoor vector control interventions against malaria from mosquito data
Source: Nat Commun. 2022 Jul 5;13:3862. doi: 10.1038/s41467-022-30700-1 (PMC9256631; doi:10.1038/s41467-022-30700-1)
Supplement: Supplementary file 1 — Supplementary Information [file 41467_2022_30700_MOESM1_ESM.pdf]

Inferring the epidemiological benefit of indoor vector control interventions against malaria from mosquito data

Authors: E Sherrard-Smith[1], Corine Ngufor[2,3], Antoine Sanou[4], Moussa Guelbeogo[4], Raphael N'Guessan[5,3], Eldo Elobolobo[6], Francisco Saute[6], Kenyssony Varela[7], Carlos Chaccour[8], Rose Zulliger[9], Joe Wagman[10], Molly L. Robertson[10], Mark Rowland[3], Martin Donnelly[11], Samuel Gonahasa[12], Sarah Staedke[3], Jan Kolaczinski[13], Thomas S. Churcher*[1].

SUPPLEMENTARY INFORMATION

<u>This document contains:</u>

<u>1. Supplementary Methods</u>
<u>2. Supplementary Figures 1-19</u>
<u>3. Supplementary Tables 1-4</u>

Supplementary Methods

Quantifying the efficacy of ITNs

Two functions can be reasonably used to capture the association between mosquito mortality induced by pyrethroid nets in experimental huts (l_1) and the susceptibility bioassay (l_b). We explore the predictive power of both functions and repeat the analysis from Nash et al.[1] here for clarity. The logistic growth function describes the data such that:

$$l_1 = \frac{1}{\left(1+exp^{(-(l_b-\beta 2a)\times\beta 1a)}\right)} \qquad (S1.1)$$

The parameters β_{1a} and β_{2a} determine the shape of the relationship (Equation S1.1) and are distinct if we fit to the combined West and East African hut data, or to each resource separately (Supplementary Table 2).

The alternative log-logistic function indicates that the association between mortality metrics can be captured by:

$$l_1 = \frac{1}{\left(1+\left(\frac{l_b}{\beta 1b}\right)^{-\beta 2b}\right)} \qquad (S1.2)$$

This time, the parameters β_{1b} and β_{2b} determine the shape of the relationship (Equation S1.2) and are once again distinct if we fit to the combined West and East African hut data, or to each resource separately (Supplementary Table 2).

For simplicity we assume that all pyrethroid-only nets have the same entomological impact (for example, conventional nets dipped in pyrethroid insecticide are the same as long-lasting insecticidal nets which incorporate or coat the pyrethroid insecticide inside the fabric during the manufacturing process. The added benefit of nets with the synergist piperonyl butoxide (pyrethroid-PBO) ITNs is determined by a meta-analysis of EHT data (in press) to determine the association between mosquito mortality induced in 24-hours by pyrethroid-only nets in experimental huts (l_1) and the corresponding mortality induced by the presence of pyrethroid-PBO ITNs (l_2):

$$l_2 = \frac{1}{\left(1+exp^{-(\omega_1+\omega_2(l_1))}\right)} \,. \qquad (S1.3)$$

The parameters ω_1 and ω_2 determine the shape of the association (Equation S1.3) (Supplementary Table 2).

Any given *Anopheles* mosquito feeding attempt is assumed to result in either mosquito mortality, successful blood-feeding, exiting unfed, or being deterred without entering the house[1]. The probability of mosquitoes entering a hut with a pyrethroid-only net relative to one without interventions present can be described such that:

$$det = \partial_3 \times exp^{\left(\delta_2\times\left(1-exp^{(\partial_1\times l_p)}\right)\right)} \qquad (S1.4)$$

Here the proportion of mosquitoes deterred from the hut (*det*) is associated with the induced mortality from the treated net that is specific to the net type deployed (p = 1 for pyrethroid-

only nets (or CTNs) and p = 2 for pyrethroid-PBO ITNs). The parameters $\delta_{1\text{-}3}$ describe the shape of the association.

Mosquitoes entering, blood-feeding and surviving (*fed*) are associated such that:

$$fed = 1 - \exp\,(\rho_1 \times \frac{(1-\exp(\rho_2 \times lp))}{\rho_2}) \qquad \text{(S1.5)}$$

Again, the parameters, ρ_1 and ρ_2 that describe the association with mortality given the net type deployed, are noted in Supplementary Table 2.

We assume that the local mosquitoes are entirely represented through the data collated in experimental huts such that there are only 3 probable outcomes from a mosquito feeding attempt in the presence of a treated net; mosquitoes can be killed (l_p), successfully feed and leave (k_p) or do neither and repeat (j_p). The estimates for j_p, k_p, and l_p are associated following Griffin et al.[3]:

$$r_{p0} = \left(1 - \frac{k'_p}{k_0}\right)\left(\frac{j'_p}{j'_p + l'_p}\right) \qquad \text{(S1.6)}$$

$$d_{p0} = \left(1 - \frac{k'_p}{k_0}\right)\left(\frac{l'_p}{j'_p + l'_p}\right) \qquad \text{(S1.7)}$$

$$s_{p0} = \frac{k'_p}{k_0} \qquad \text{(S1.8)}$$

Where $j_p' = m_p j_p + (1 - m_p)$, $k_p' = m_p k_p$, and $l_p' = m_p l_p$ [3]. Historical values are used to estimate k_0; the mosquitoes that enter a house and successfully feed in the absence of an intervention [4–6] (Supplementary Table 2).

In the absence of an alternative data resource, we estimate waning efficacy of the active ingredient in treated nets over time from EHT data on washed nets. A durability study provides a prior estimate for treated net half-life in years for pyrethroid-only nets[7]. The half-life of the killing activity of the pyrethroid in ITNs (H_y) is therefore assumed to be proportional to the lost morbidity accumulated through washing ITNs 20 times (half-life in washes, H_W). Pyrethroid resistance in local mosquitoes may also increase the waning of the active ingredient over time. To reflect changes driven by pyrethroid resistance, $H_y = H_w/H_p^s H_Y^s$, where superscript s shows pyrethroid-net half-life in a fully susceptible *Anopheles* population. Following Griffin et al. [3], insecticide activity is assumed to decay at a constant rate given γ_p, the decay parameter, which relates to half-life such that $H_W = \ln(2)/\gamma_p$. Treated net induced mortality from any ITN is then assumed to be:

$$logit(\gamma_p) = \mu_p + \rho_p(l_p - \tau) \qquad \text{(S1.9)}$$

Shape parameters μ_p, and ρ_p are assumed to be consistent between net types[2] however, it should be noted that the durability of pyethroid-PBO ITNs under natural conditions in the field has not currently been evaluated so it is unknown whether the 20 washes still applies. This should be verified in EHTs as a matter of urgency.

Entomological parameterization of IRS products

The different impacts of IRS products have been determined from a systematic review of experimental hut data [8]. Briefly, for each product a flexible logistic function is fitted to capture changing IRS impact over time since implementation (t, in days),

$$l_S = \frac{1}{1+\exp\left(-\left(l_{S\vartheta}+l_{S\gamma}\times t\right)\right)} \tag{S1.10}$$

$$N_{dead} \sim binomial(l_s, N_{total1}) \tag{S1.11}$$

$$k_S = \frac{k_0}{1+\exp\left(-\left(k_{S\vartheta}+k_{S\gamma}\times t\right)\right)} \tag{S1.12}$$

$$N_{successfully\_fed} \sim binomial(k_s, N_{total1}) \tag{S1.13}$$

$$m_S = \frac{1}{1+\exp\left(-\left(m_{S\vartheta}+l_{S\gamma}\times t\right)\right)} \tag{S1.14}$$

$$N_{deterred} \sim binomial(m_s, N_{total2}) \tag{S1.15}$$

Parameter l_S denotes the proportion of mosquitoes being killed given initial efficacy ($l_{S\vartheta}$) and impact duration ($l_{S\gamma}$). The logistic model is fitted using the total number of mosquitoes killed (N_{dead}) in the sprayed huts (N_{total1}). The proportion of mosquitoes successfully feeding (k_S) and being deterred away from a sprayed hut (m_S) are similarly determined. Deterred mosquitoes ($N_{deterred}$) are calculated from the difference between mosquitoes in control and sprayed huts. The proportion of mosquitoes that enter and are then repelled without being killed or feeding is then $j_s = 1 - l_s - k_s$. Previously the parameters for the transmission model have been fitted and estimate k_0 as 0.699 [4,5]. The k_s fits are scaled to ensure that the probabilities that a mosquito entering a sprayed hut successfully blood-feeds, exits without feeding or dies, denoted s_S, r_S and d_S respectively, are within the 0 to 1 range. For non-pyrethroid IRS products, the parameter estimates are given in Data S1.5).

The association between mosquito mortality measured in discriminatory dose bioassays and mortality induced by IRS in experimental huts is also used to estimate the diminishing impact of pyrethroid-based IRS with resistance [8]. Briefly, the mosquito mortality ($l_{S\,(t=0)}$), blood-feeding ($k_{S\,(t=0)}$) and deterrence ($m_{S\,(t=0)}$) measured immediately after spraying pyrethroid insecticide on the walls of an experimental hut can be associated with bioassay survival (as a measure of resistance, x) as follows:

$$l_{S(t=0)} = \frac{1}{1+\exp\left(-\left(\mu_{S\vartheta}+\mu_{S\gamma}\,x\right)\right)}, \tag{S1.16}$$

$$N_{dead} \sim binomial\left(l_{s(t=0)}, N_{total1}\right)$$

$$k_{S(t=0)} = \frac{k_0}{1+\exp\left(-\left(\beta_{S\vartheta}+\beta_{S\gamma}\,x\right)\right)}, \tag{S1.17}$$

$$N_{successfully\_fed} \sim binomial\left(k_{s(t=0)}, N_{total1}\right)$$

$$m_{S(t=0)} = \frac{1}{1+\exp\left(-\left(\varepsilon_{S\vartheta}+\varepsilon_{S\gamma}\,x\right)\right)}. \tag{S1.18}$$

$$N_{deterred} \sim binomial\left(m_{s(t=0)}, N_{total1}\right)$$

The change in mosquito mortality (l_S), blood-feeding (k_S) and deterrence (m_S) observed over time (Equations S1.11 – S1.16) are estimated for each experimental hut study separately to generate 21 parameter sets (unique studies in [8] on pyrethroid IRS) describing how each trait changes over time. The parameters k_0 defining successfully fed mosquito in the absence of indoor interventions was previously estimated as 0.699 [3] so k_s fits are scaled to return probabilities that are within the appropriate 0 to 1 range. These parameters are then associated with $t = 0$ mortality, blood-feeding and deterrence such that,

$$l_{S\vartheta} = \alpha_{l1} + \alpha_{l2} l_{S(t=0)}, \tag{S1.19}$$

$$l_{S\gamma} = \alpha_{l3} + \alpha_{l4} l_{S(t=0)}, \tag{S1.20}$$

$$k_{S\vartheta} = \alpha_{k1} + \alpha_{k2} k_{S(t=0)}, \tag{S1.21}$$

$$k_{S\gamma} = \alpha_{k3} + \alpha_{k4} k_{S(t=0)}, \tag{S1.22}$$

$$m_{S\vartheta} = \alpha_{m1} + \alpha_{m2} m_{S(t=0)}, \tag{S1.23}$$

$$m_{S\gamma} = \alpha_{m3} + \alpha_{m4} m_{S(t=0)}, \tag{S1.24}$$

where α_{l1} to α_{l4} determine how initial mosquito mortality $l_{s\vartheta}$ and the duration of impact $l_{s\gamma}$ are altered by increasing pyrethroid resistance. Similarly, for $k_{S\vartheta}$, $k_{S\gamma}$, α_{k1-4} or $m_{S\vartheta}$, $m_{S\gamma}$, α_{m1-4} enables the impact of pyrethroid resistance on successful blood-feeding or deterrence to be quantified. Substituting in Equations S1.20, S1.21 to Equation S1.11 (and so on for S1.22 – S1.24) enables the relationships between prevalence, pyrethroid resistance and the change in IRS efficacy over time to be characterized for pyrethroid IRS (Data S1.5). We can then use the bioassay mortality data from respective trial arms to estimate an entomological impact of a pyrethroid-based IRS interventions.

Modelling uncertainty

Uncertainty bounds measured for data recorded during the RCTs were used to generate 1000 parameter estimates for the model simulations. Where data estimates were bound between 0 and 1 we assumed a beta distribution to generate uncertainty, where appropriate we assumed a normal distribution for trial data estimates, always ensuring the range fell within that observed by the trials. Uncertainty was also incorporated from the entomological impacts estimated from EHT data and bioassay data testing mosquito susceptibility to pyrethroid insecticides [2,8]. Where RCT data were not available to inform parameter estimates, default estimates were used as noted in Data S6. The full input parameter sets for each study and each trial arm are provided in https://github.com/EllieSherrardSmith/ibm_rct_prediction.

We do not include uncertainty in our projections that are derived from the model fitting. Instead, we use the median draw from the posterior predictions of model parameters. This is because we are principally interested, in this sensitivity analysis, in the uncertainty driven by vector control interventions and RCT data. We have shown previously the uniform nature of uncertainty from model parameters [9] which would be consistent across simulations but would dilute uncertainty from ITN and IRS parameters and increase the computational effort.

The model is calibrated individually to the baseline prevalence in a defined age cohort for a total of 37 trial arms from 13 RCTs (Supplementary Table 1), observed malaria prevalence for

each trial shown in Figures S4-S16. Malaria prevalence in the different arms is then simulated moving forward using only baseline parameters, the timing of vector control interventions (including repeated deployment of IRS) and the rate of loss of ITN use over time (as estimated for each trial).

Further assumptions

The model considers average bionomics for mosquito species that remain constant throughout the year so does not account for any potential changes in behavior with temperature or season. The method uses average trends in mosquito survival during bioassay testing which is a test with highly variable results[10]. EHT data are also variable due to differences in the size of the huts, wall surfaces, baffle size, shape and location, hut wall and ceiling surface and the intervention provided for the control hut sleeper (e.g. no net, a holed untreated net)[11]. Our process assumes that taking the average of these data can smooth some of the variation observed in different locations to generalize the effects of the tested products.

There is a lack of evidence of the existence of pyrethroid-resistance mosquitoes for the early trials. In the absence of this early bioassay data it is assumed that studies conducted prior to 2000 all mosquito species were susceptible to pyrethroid. This matches trends seen elsewhere in the continent [WHO: Malaria Threats Map][12] but the results of these trials should be treated with caution to reflect this. We assume no insecticide resistance to non-pyrethroid products throughout this work.

The model is calibrated by varying the ratio of mosquitoes to humans until the baseline burden of malaria matches that observed point estimate of malaria prevalence at baseline for each trial arm. To avoid over fitting the model, in this validation exercise, we match the average seasonality of the district given the past 10-years of rainfall data and introduce a 1-month lag to allow mosquito peak abundance to be recovered. This makes the predictions less specific to the weather patterns of the region for the specific year(s) of the trials but means that we can confirm we remain able to make robust predictions in the absence of knowing exact weather patterns driving transmission. The percentage of cases diagnosed with a rapid diagnostic test (RDT) and promptly treated with the recommended treatment for the region will also change the trajectory of the prediction to some degree and this will be more significant for smaller trials where this cohort represents a greater proportion of the total community. However, we make the simplifying assumption that the active cohort tracked during the trials does not alter the population-level dynamics.

Supplementary Figures

Supplementary Figure S1.

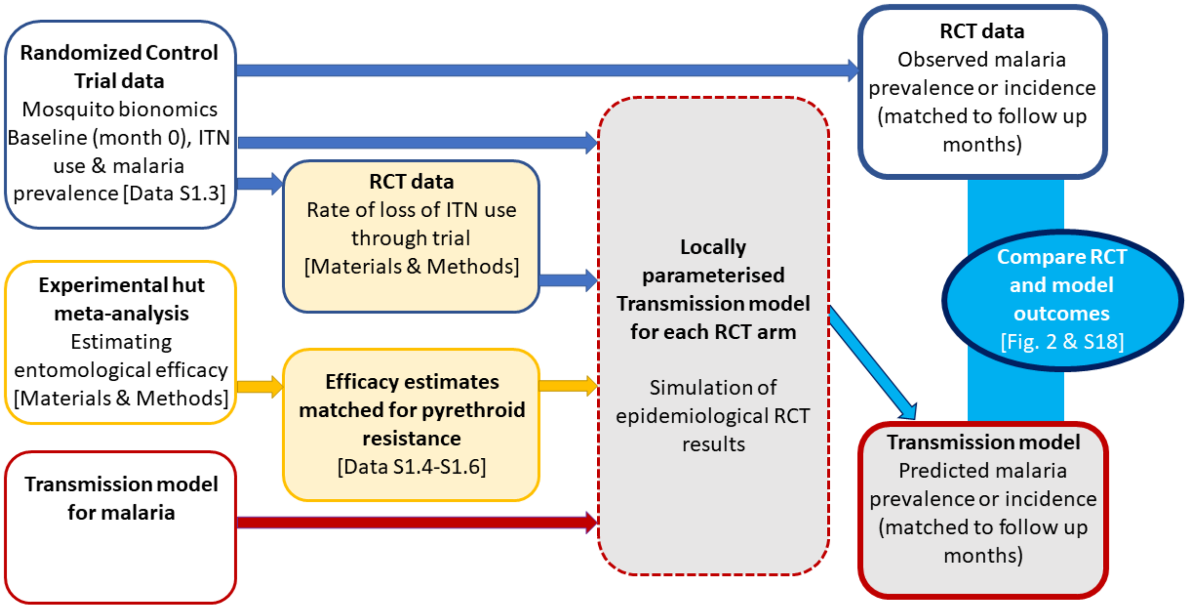

A schematic of the simulation process for each cluster randomized trial arm and how it is compared to observed data from the trial. The boxes highlight and sources of data included in the Supplementary Information and refer to Figures in the main text or supplement.

Supplementary Figure S2.

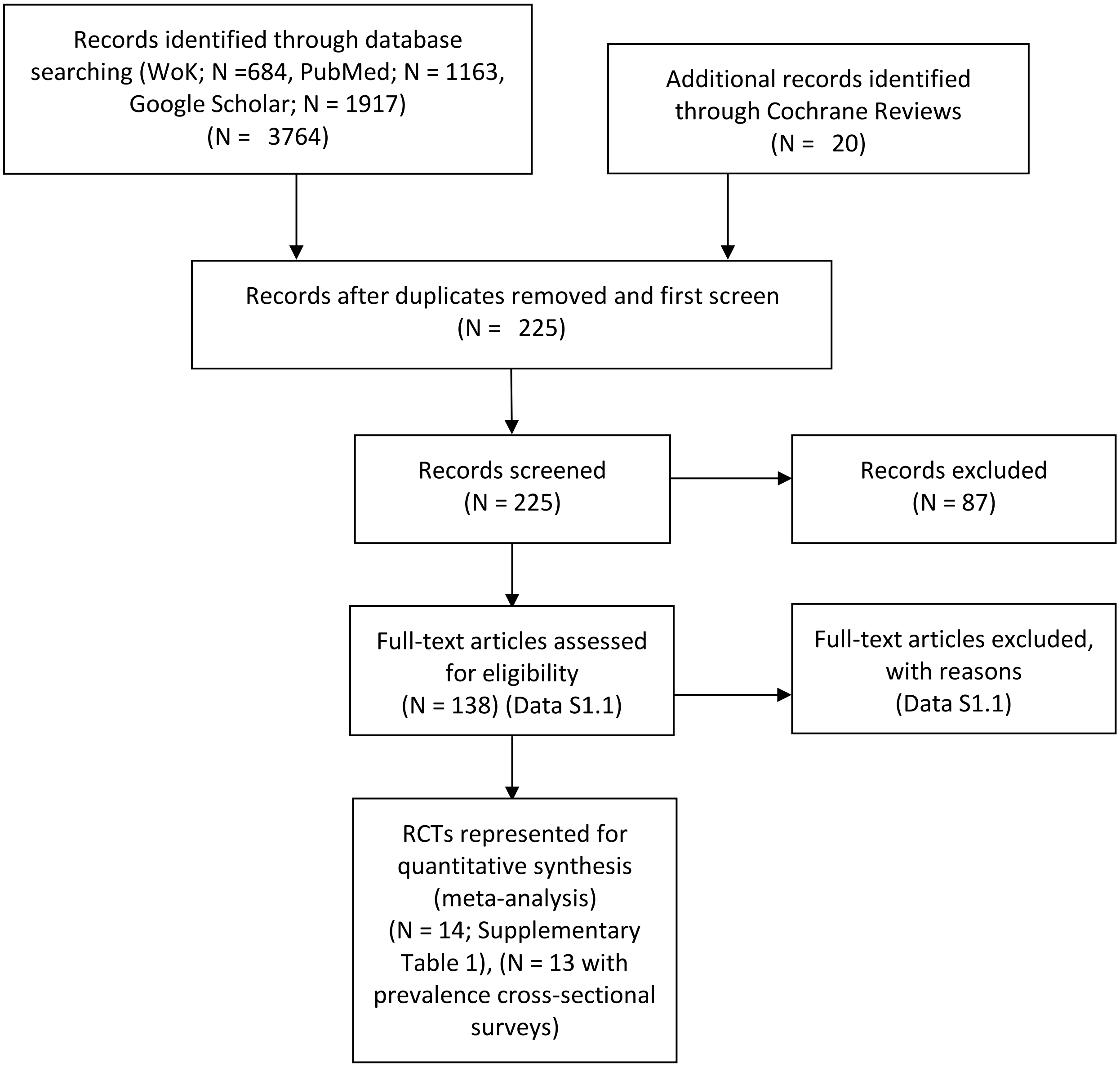

Schematic of the systematic review to identify appropriate cluster randomized control trials to assess in full.

Supplementary Figure S3-S15 The following set of figures show the model predictions of the best-fit model (option 4, Supplementary Table 3) to the observed data for the individual trials. Points (with 95% confidence intervals where data are available) show observed data recorded in the trial whilst solid horizontal timelines indicate model predictions. In all plots the trial is summarized in the caption and the models presented are for model 4 (Supplementary Table 3) which assumes the relationship between bioassay and hut trial mortality is explained by the log-logistic function and ITN and IRS performance parameters are characterized by combining data from all experimental hut trials. Specific parameters are provided in Supplementary Data S1.4 - S1.6.

Supplementary Figure S3

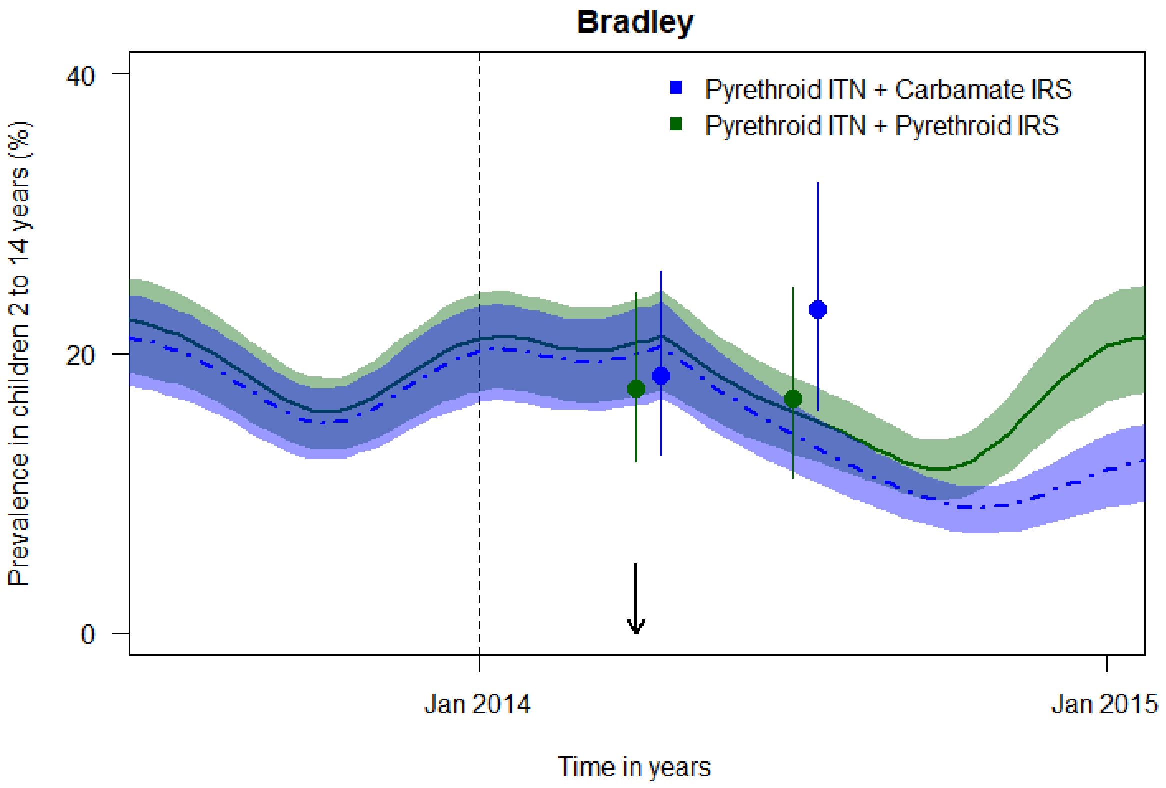

Bradley et al. 2016 [13]; Bioko Island, Equatorial Guinea; IRS is implemented, addition to background distributions of pyrethroid nets (brand unknown). Arm 1 tests the addition of carbamate (Ficam®) IRS and arm 2 tests the deltamethrin-based pyrethroid IRS (March-April 2014). The model simulated RDT-prevalence over time for children aged 2 – 14 years is shown as a solid light blue line (carbamate IRS) or dark green line (pyrethroid IRS) with uncertainty in parameter estimates included for ITN and IRS efficacy and for data recording uncertainty, observed in the trial and used for parameter estimation. Dashed vertical line indicates the baseline time for prevalence observations while arrows indicate the time when interventions are distributed. Points indicate trial observed prevalence for children aged 2 – 14 years. Vertical coloured lines show 95% confidence interval estimates reported by the original study (Supplementary Data S1.3).

Supplementary Figure S4

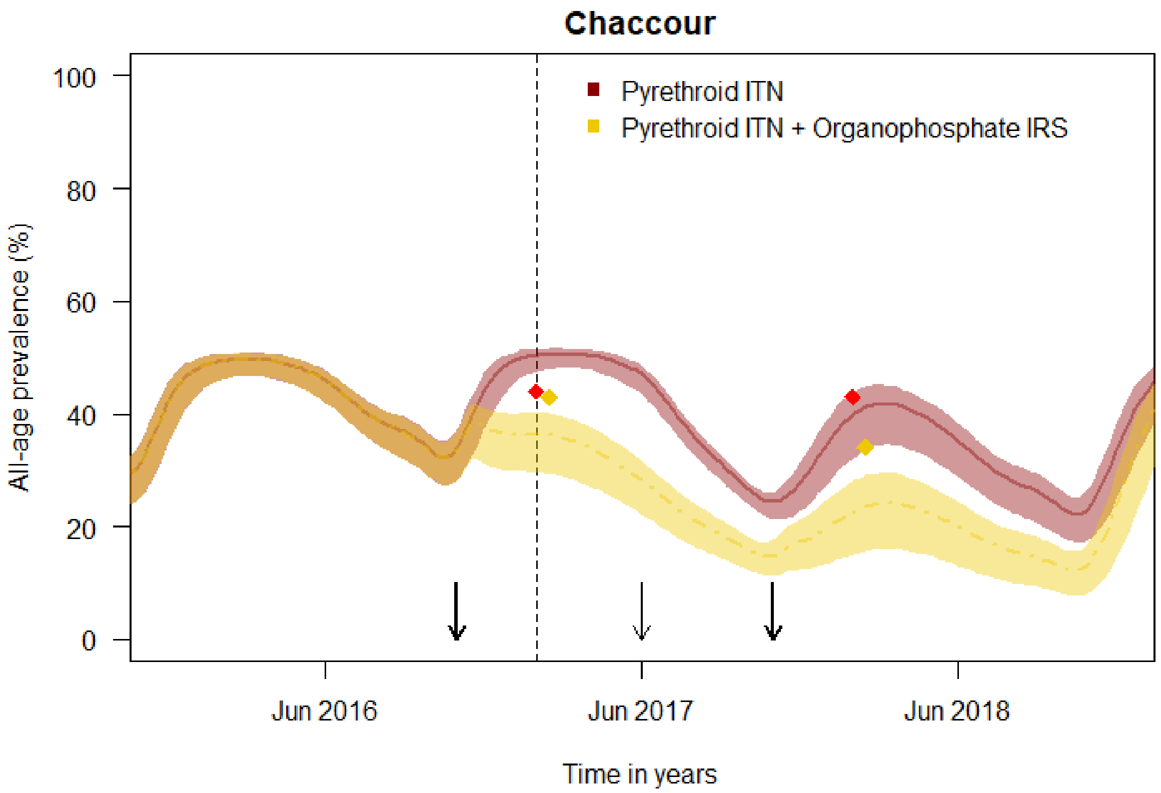

Chaccour et al[14]; Zambesia, Mozambique; IRS is implemented, additional to background distribution of ITNs (PermaNet 2.0). Arm 1 measures pyrethroid nets alone and arm 2 measures the addition of Actellic® 300CS implemented in October-November 2016 and 2017. A mass ITN distribution was completed in 2017. The model simulated RDT-prevalence over time for children under 5 years is shown as a solid red line (net only arm) or yellow line (Actellic® 300CS IRS). Dashed vertical line indicates the baseline time for prevalence observations (in this RCT the first estimate for prevalence was made in February 2017 after implementation of the IRS) while arrows indicate the time when interventions are distributed. Points indicate trial observed prevalence for children under 5 years.

Supplementary Figure S5

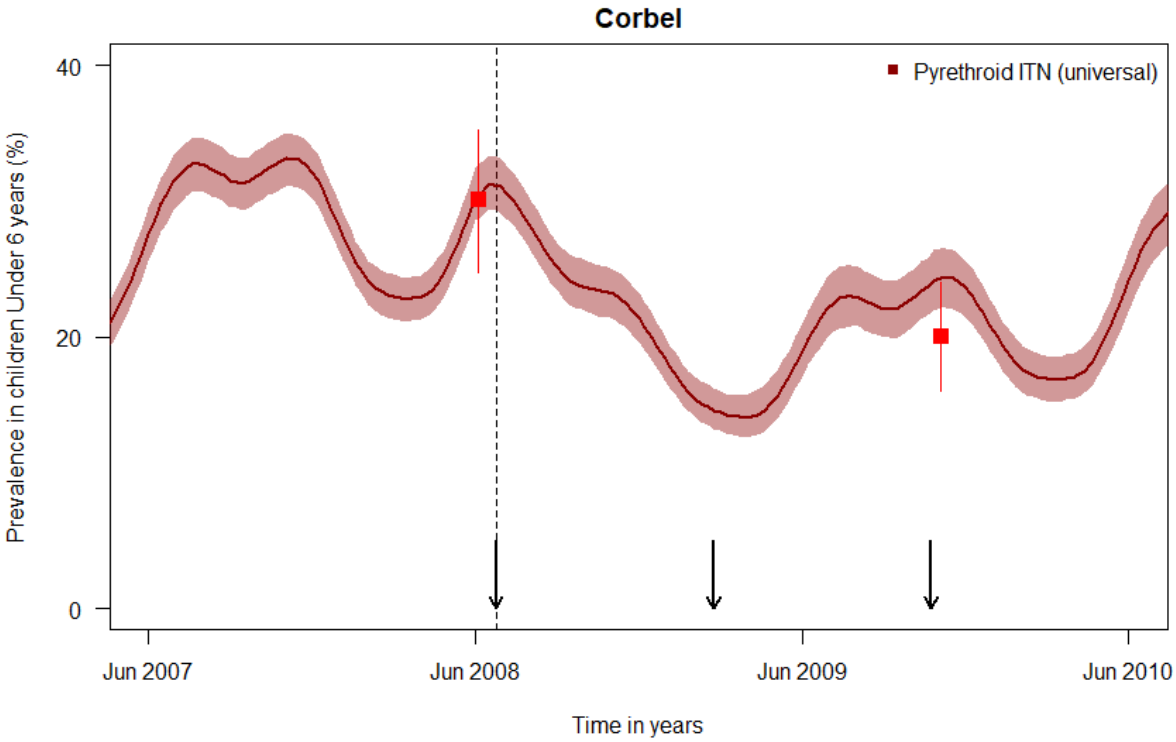

Corbel et al 2012[15]; Ouidah-Kpomassè-Tori Health Area, Southern Benin, Benin; A 4 arm trial was conducted but carbamate sprayed curtains have not been parameterized using entomological data from experimental huts, so this arm is excluded. PermaNet 2.0 pyrethroid nets are targeted to pregnant women and children (arm 1), used universally (arm 2), or targeted to pregnant women and children and used together with carbamate IRS Ficam® (arm 3). Interventions are implemented from June 2008 onward and monitored after 18 months. The model is set up for random distribution so arms targeting net use to pregnant women and children are excluded. The universal net distribution trial arm is shown. The model simulated RDT-prevalence over time for children under 6 years is shown as a solid red line (universal ITNs). Dashed vertical line indicates the baseline time for prevalence observations while arrows indicate the time when interventions are distributed. Points indicate trial observed prevalence for children under 5 years. Vertical coloured lines show 95% confidence interval estimates reported by the original study (Supplementary Data S1.3).

Supplementary Figure S6

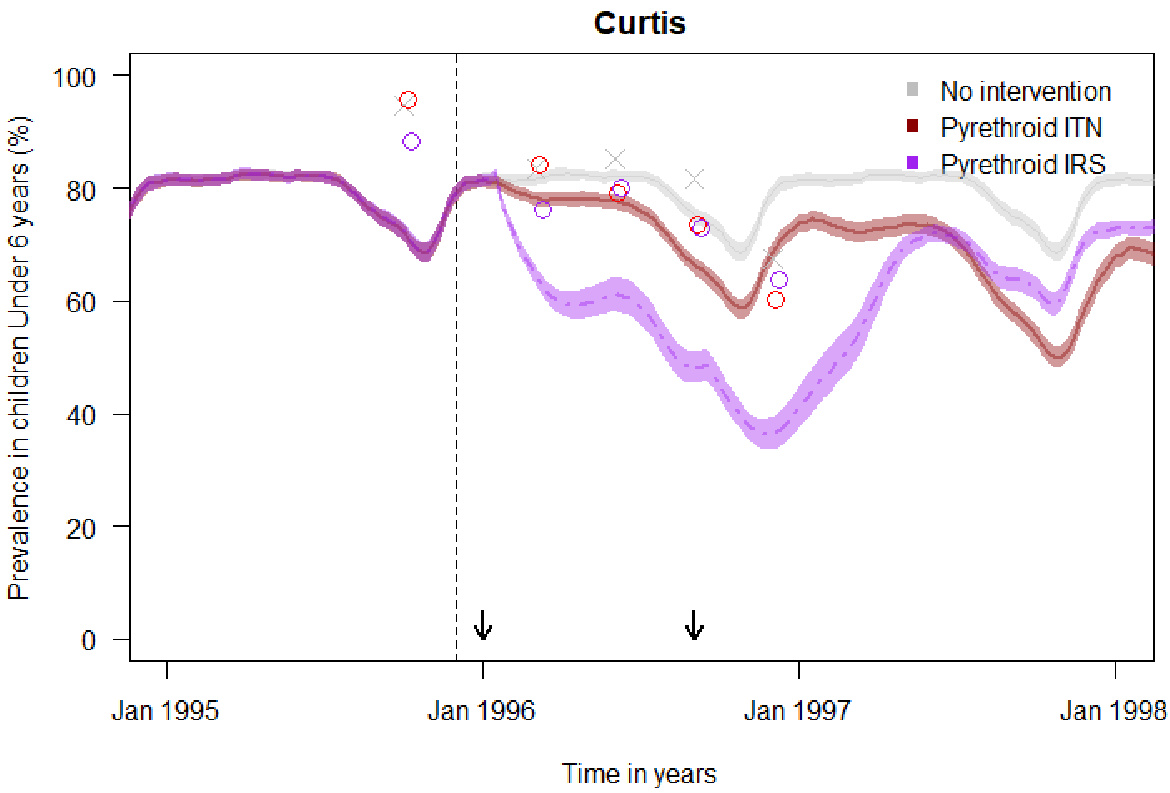

Curtis et al 1998[16]; Muheza, Tanga Region, Tanzania; No interventions are provided to the first cluster (arm 1), conventional dipped nets (CTNs) are given to the second cluster (arm 2) and Icon CS 10% IRS (lambdacyhalothrin) was used in the 3rd cluster (arm 3). The RCT began in December 1995 and is monitored for 11 months. The model simulated RDT-prevalence over time for children 1 to 6 years old is shown as a solid light grey line (no interventions), red line (CTNs) or blue line (Icon CS 10% IRS). Dashed vertical line indicates the baseline time for prevalence observations while arrows indicate the time when interventions are distributed. Points indicate trial observed prevalence for children 1 to 6 years old.

Supplementary Figure S7

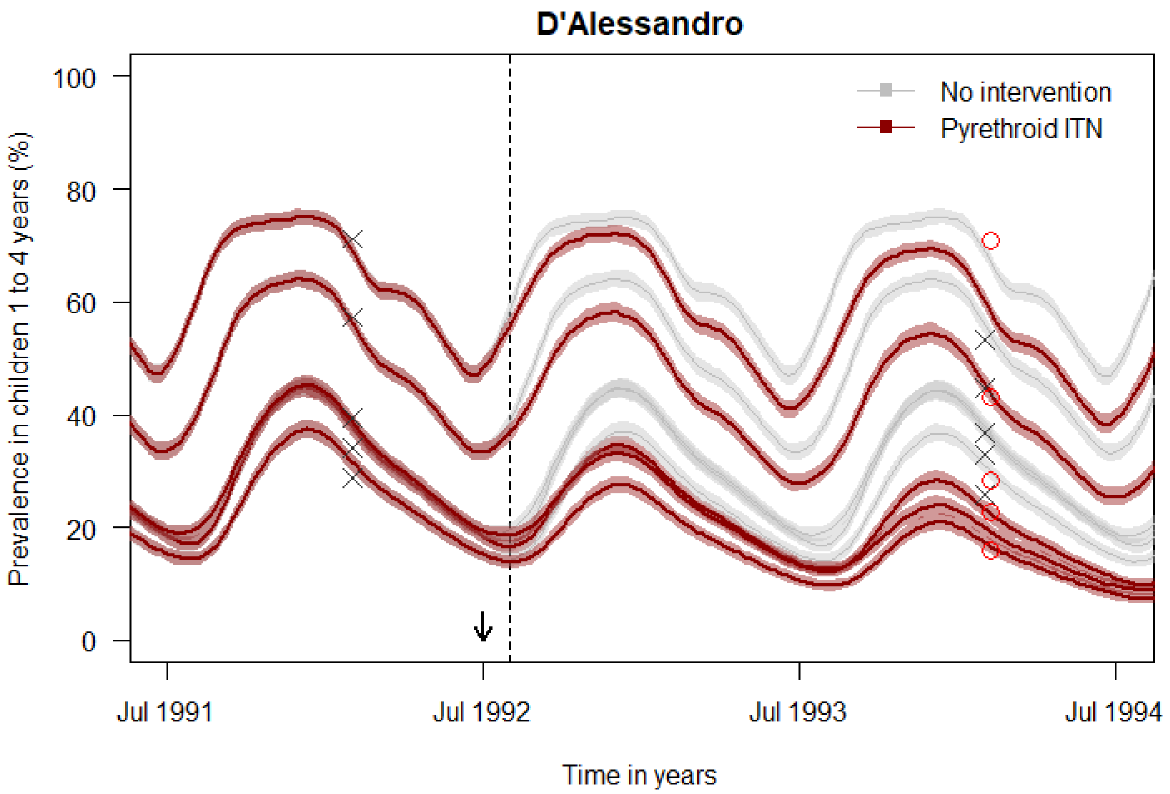

D'Alessandro et al 1995[17]; The Gambia; Five villages have untreated nets and five villages have conventionally treated dipped nets (CTNs), these settings are reported to have matched prevalence at baseline (Data S1.3). The settings are spread throughout The Gambia. The trial is initiated in July 1992. The model simulated RDT-prevalence over time for children 1 to 4 years old is shown as solid light grey lines (untreated nets), or red lines (CTNs). Dashed vertical line indicates the baseline time for prevalence observations while arrows indicate the time when interventions are distributed. Points indicate trial observed prevalence for children 1 to 4 years old.

Supplementary Figure S8

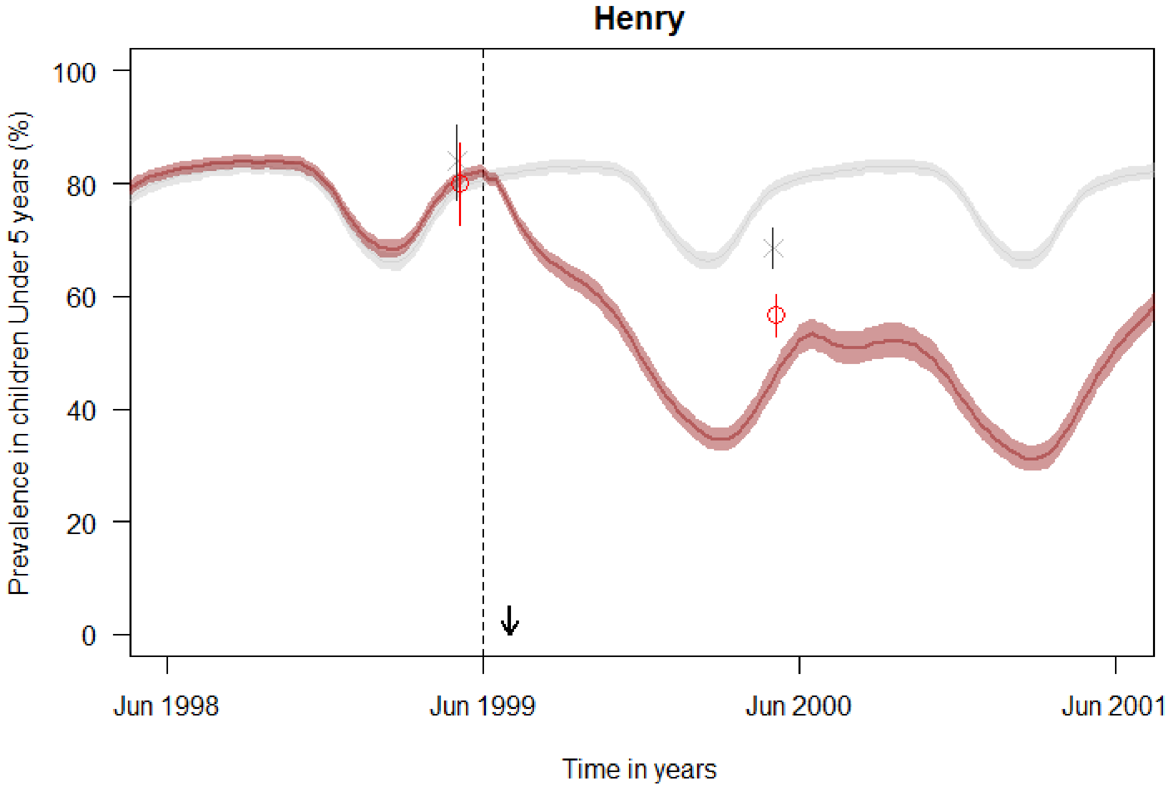

Henry et al 2005[18]; Korhogo, Savanes, Côte D'Ivoire; This RCT compares untreated nets with conventionally treated pyrethroid dipped nets (CTNs, brand unknown) distributed in June 1999 and re-dipped in December 1999. The model simulated RDT prevalence in under 5-years over time is shown as solid grey line (untreated bed nets), red line (CTNs). Dashed vertical line indicates the baseline time for prevalence observations while arrows indicate the time when interventions are distributed. Points indicate trial observed prevalence in under 5-years. Vertical solid lines show 95% confidence interval estimates reported by the original study (Supplementary Data S1.3).

Supplementary Figure S9

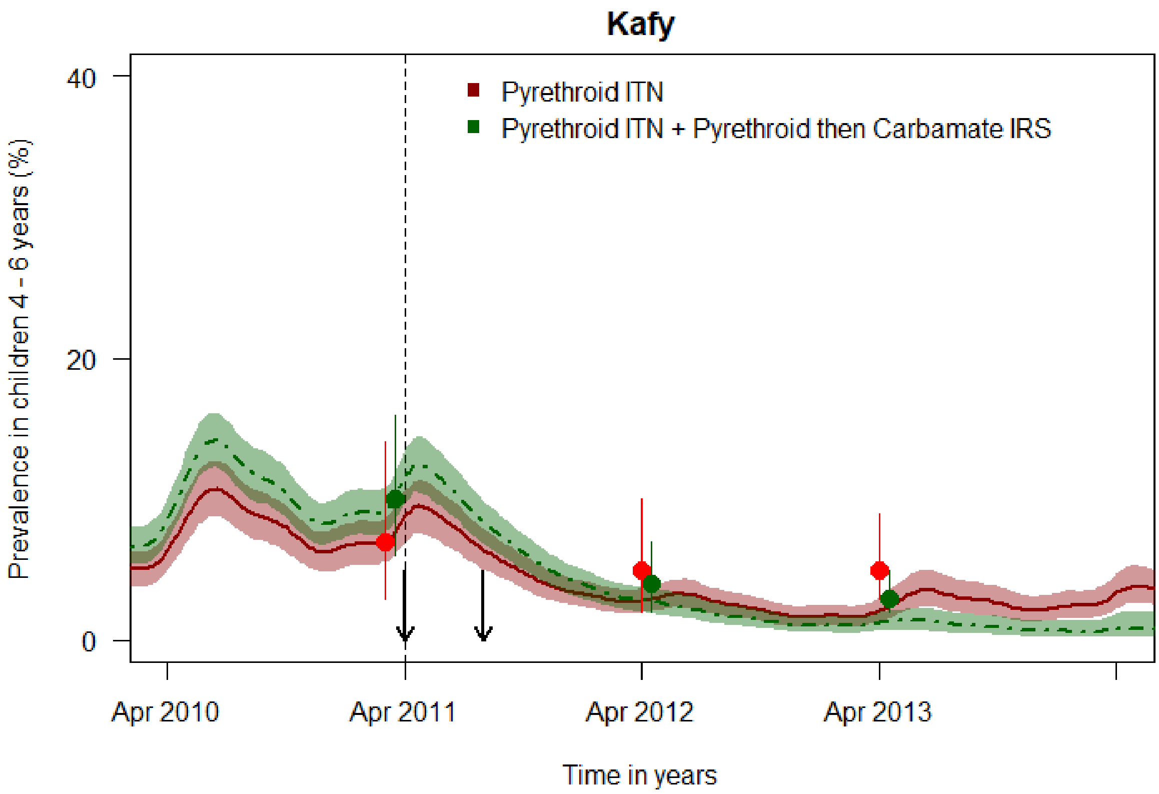

Kafy et al 2017[19]; Galabat, Sudan; PermaNet 2.0 nets with pyrethroid IRS are compared to pyrethroid nets distributed in April 2011. The model simulated RDT prevalence in 6-month to 10-year old children over time is shown as solid red line (ITNs), or blue line (ITNs and deltamethrin IRS). Dashed vertical line indicates the baseline time for prevalence observations while arrows indicate the time when interventions are distributed. Points indicate trial observed prevalence in 6-month to 10-year old children. Vertical coloured lines show 95% confidence interval estimates reported by the original study (Supplementary Data S1.3).

Supplementary Figure S10

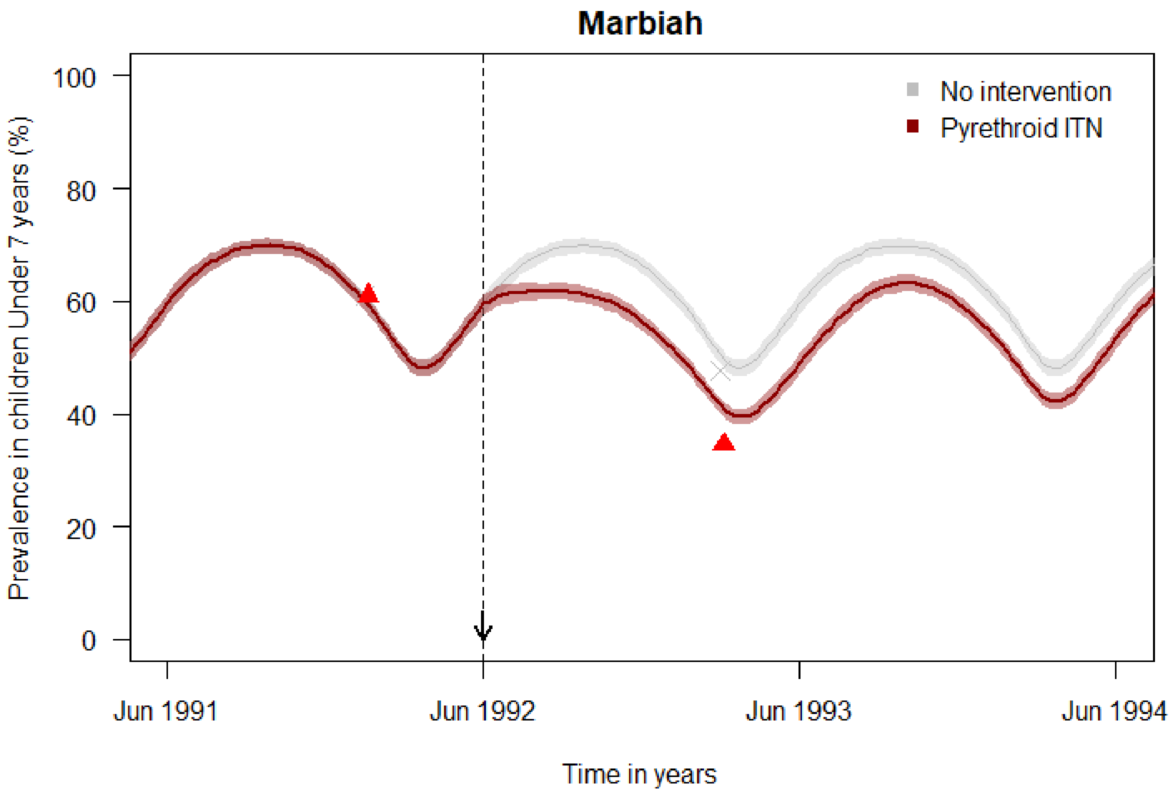

Marbiah et al 1998[20]; Bo district, Southern Province, Sierre Leone; No interventions are compared to conventional dip-nets (brand unknown). The trial is initiated in June 1992. The model simulated RDT prevalence in under 7 years over time is shown as solid grey line (no intervention), or red line (conventionally treated dip nets, CTNs). Dashed vertical line indicates the baseline time for prevalence observations while arrows indicate the time when interventions are distributed. Points indicate trial observed prevalence in under 7 years.

Supplementary Figure S11

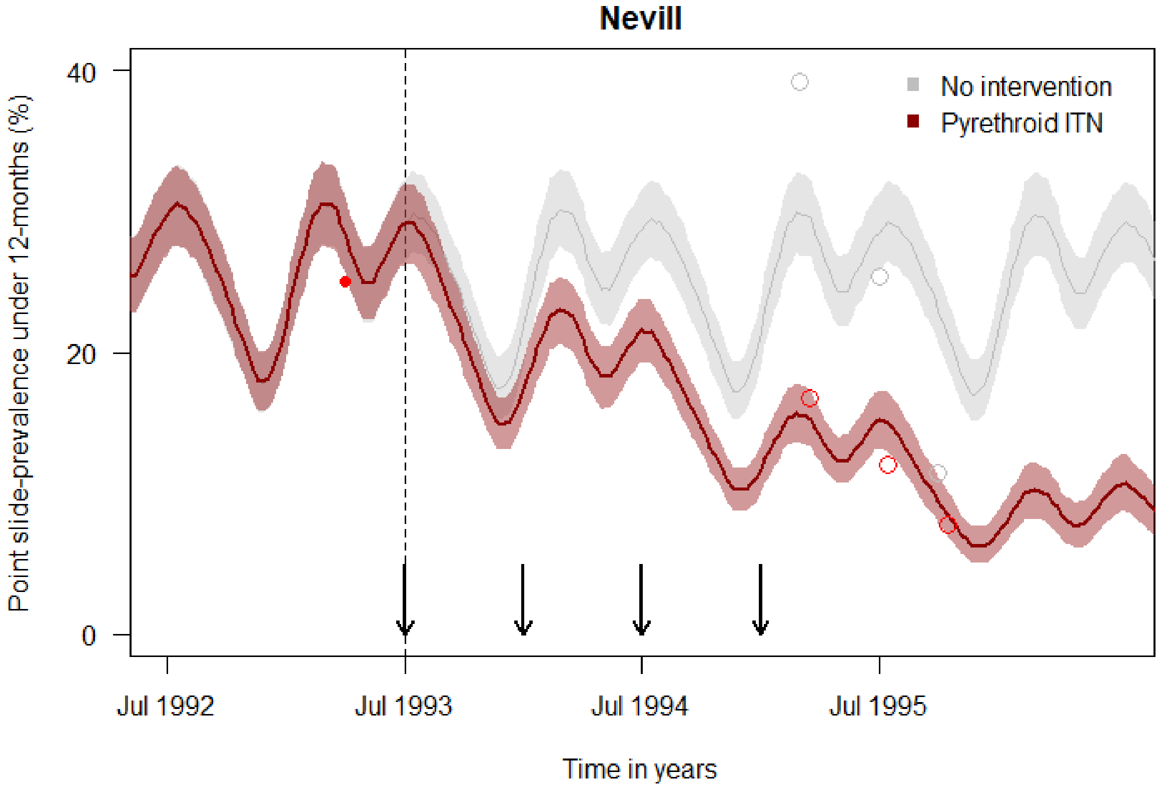

Nevill et al 1996[21]; Kilifi District, Kenya; No interventions are compared to SiamDutch conventional dip-nets CTNs. The trial is initiated in July 1993, CTNs were re-dipped bi-annually for 27 months. The model simulated RDT prevalence in under 1-year old children over time is shown as solid grey line (no intervention), or red line (CTNs). Dashed vertical line indicates the baseline time for prevalence observations while arrows indicate the time when interventions are distributed. Points indicate trial observed prevalence in under 1-year old children.

Supplementary Figure S12

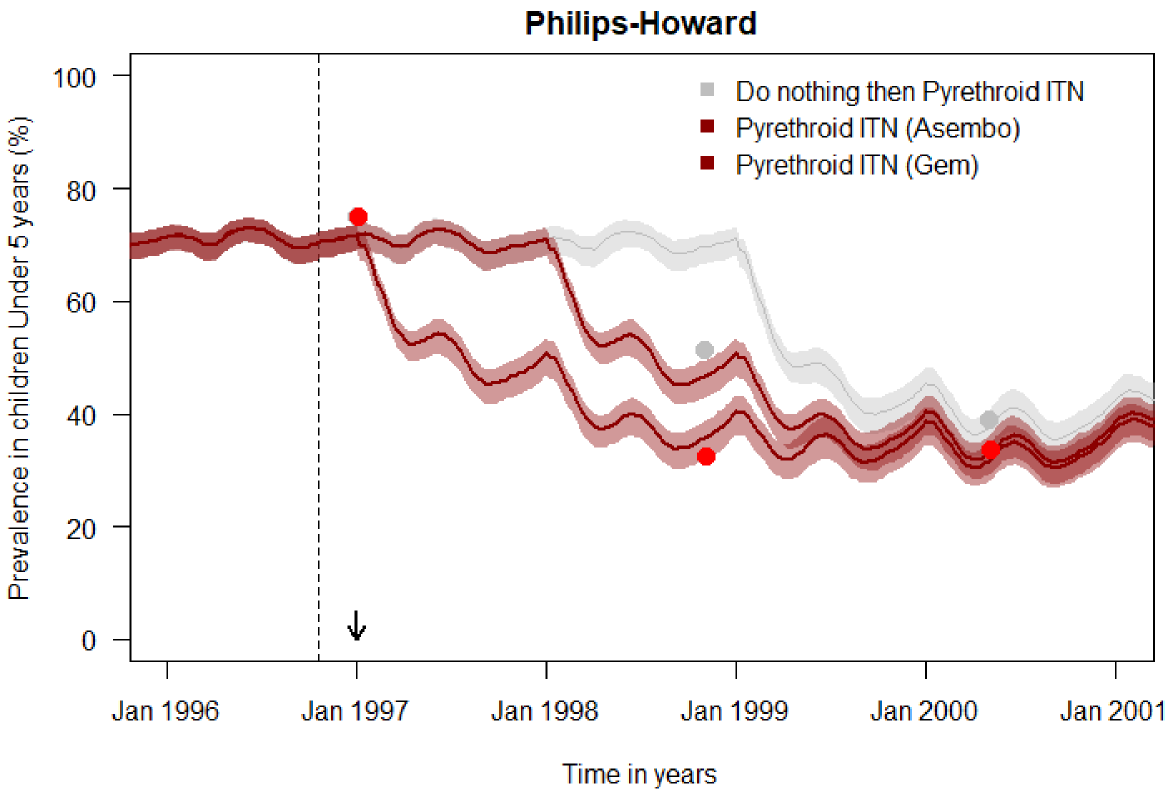

Phillips-Howard et al 2003[22]; Asembo and Gem, Western Kenya; No interventions are compared to conventionally treated dip-nets CTNs. The trial is initiated in January 1997, CTNs were re-dipped bi-annually for 30 months. The model simulated RDT prevalence in under 5-years over time is shown as solid grey line (no intervention), or red line (CTNs). Dashed vertical line indicates the baseline time for prevalence observations while arrows indicate the time when interventions are distributed. Points indicate trial observed prevalence in under 5-years.

Supplementary Figure S13

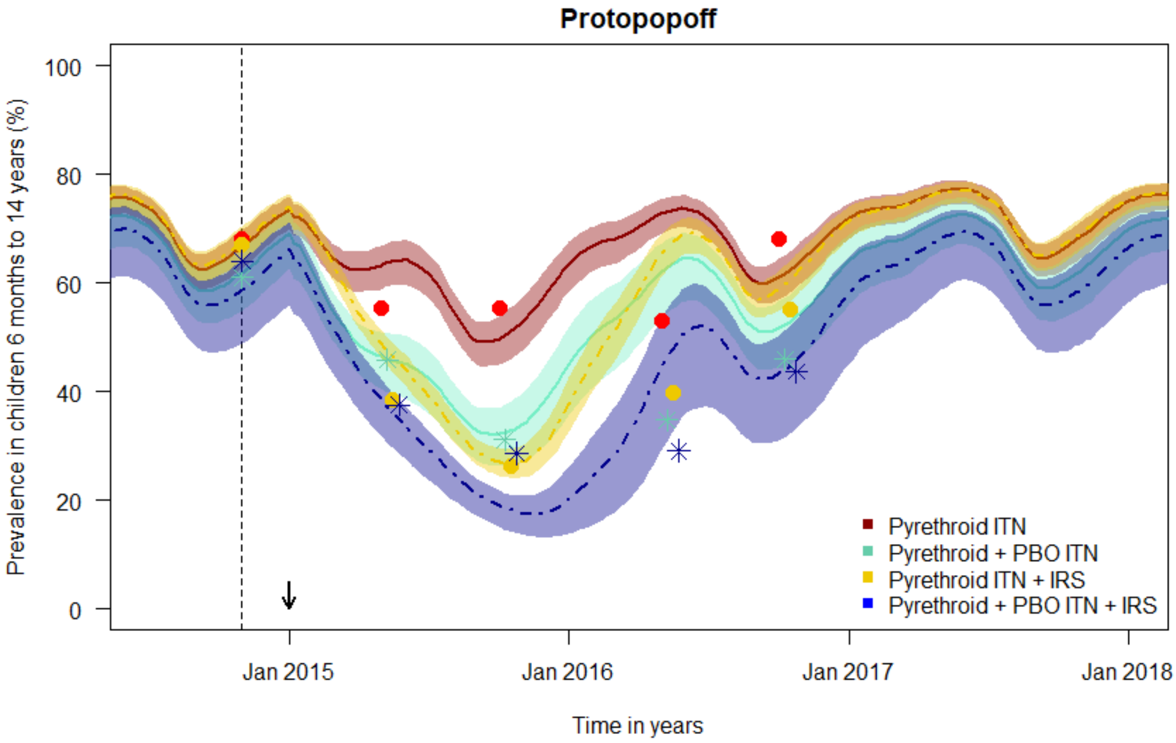

Protopopoff et al 2018[23]; Muleba district, Tanzania; Pyrethroid nets (arm 1), pyrethroid plus PBO ITNs (arm 2), pyrethroid nets with Actellic® 300CS (arm 3) and pyrethroid plus PBO with Actellic® 300CS (arm 4). Treated nets are distributed in March 2014 and Actellic® 300CS is sprayed once in March 2014. The model simulated RDT prevalence in under 6-months to 14-years over time is shown as solid red line (pyrethroid nets), purple line (pyrethroid plus PBO ITNs), blue line (pyrethroid net with Actellic® 300CS) or orange line (pyrethroid plus PBO ITNs with Actellic® 300CS). Dashed vertical line indicates the baseline time for prevalence observations while arrows indicate the time when interventions are distributed. Points indicate trial observed prevalence in 6-months to 14-years.

Supplementary Figure S14

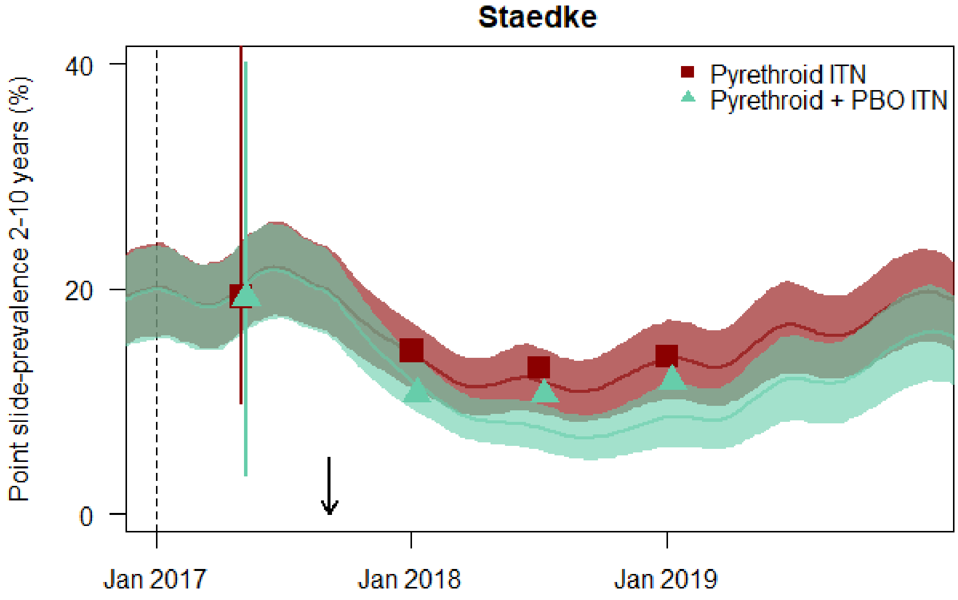

Staedke et al 2020[24]; Uganda; Pyrethroid nets (PermaNet 2.0 and Olyset Net; arm 1) and pyrethroid plus PBO ITNs (PermaNet 3.0 and Olyset Plus; arm 2) are compared. Treated nets are distributed in January 2017 across areas of Uganda without any history of IRS. The baseline prevalence is variable between arms. Only the PermaNet 2.0 and PermaNet 3.0 data are used (Olyset nets and Olyset Plus nets are also tested). The model parameterizations do not yet differentiate between product types. The model simulated RDT prevalence in 2 to 10-years over time is shown as solid red line (pyrethroid nets), purple line (pyrethroid plus PBO ITNs). Dashed vertical line indicates the baseline time for prevalence observations while arrows indicate the time when interventions are distributed. Points indicate trial observed prevalence in 2 to 10-years. Vertical coloured lines show 95% confidence interval estimates reported by the original study (Supplementary Data S1.3).

Supplementary Figure S15

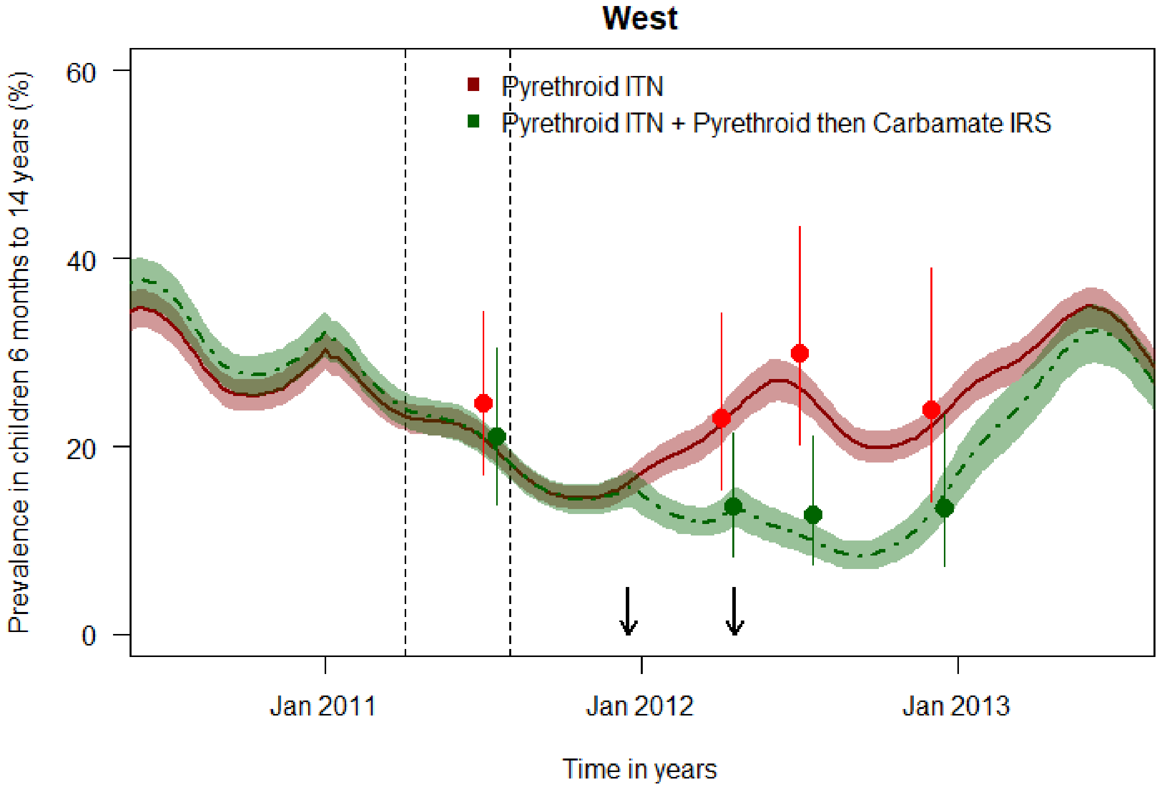

West et al 2014[25]; Muleba district, Tanzania; Pyrethroid nets (brand unknown, arm 1) and pyrethroid ITN with pyrethroid IRS used in December 2011, then carbamate IRS, Ficam®, used in April-May 2012 (arm 2) are compared. The model simulated RDT prevalence in 6-month to 14-years over time is shown as solid red line (pyrethroid nets), purple line (pyrethroid plus PBO ITNs). Dashed vertical line indicates the baseline time for prevalence observations while arrows indicate the time when interventions are distributed. Points indicate trial observed prevalence in 6-month to 14-years. Vertical coloured lines show 95% confidence interval estimates reported by the original study (Supplementary Data S1.3).

Supplementary Figure S16.

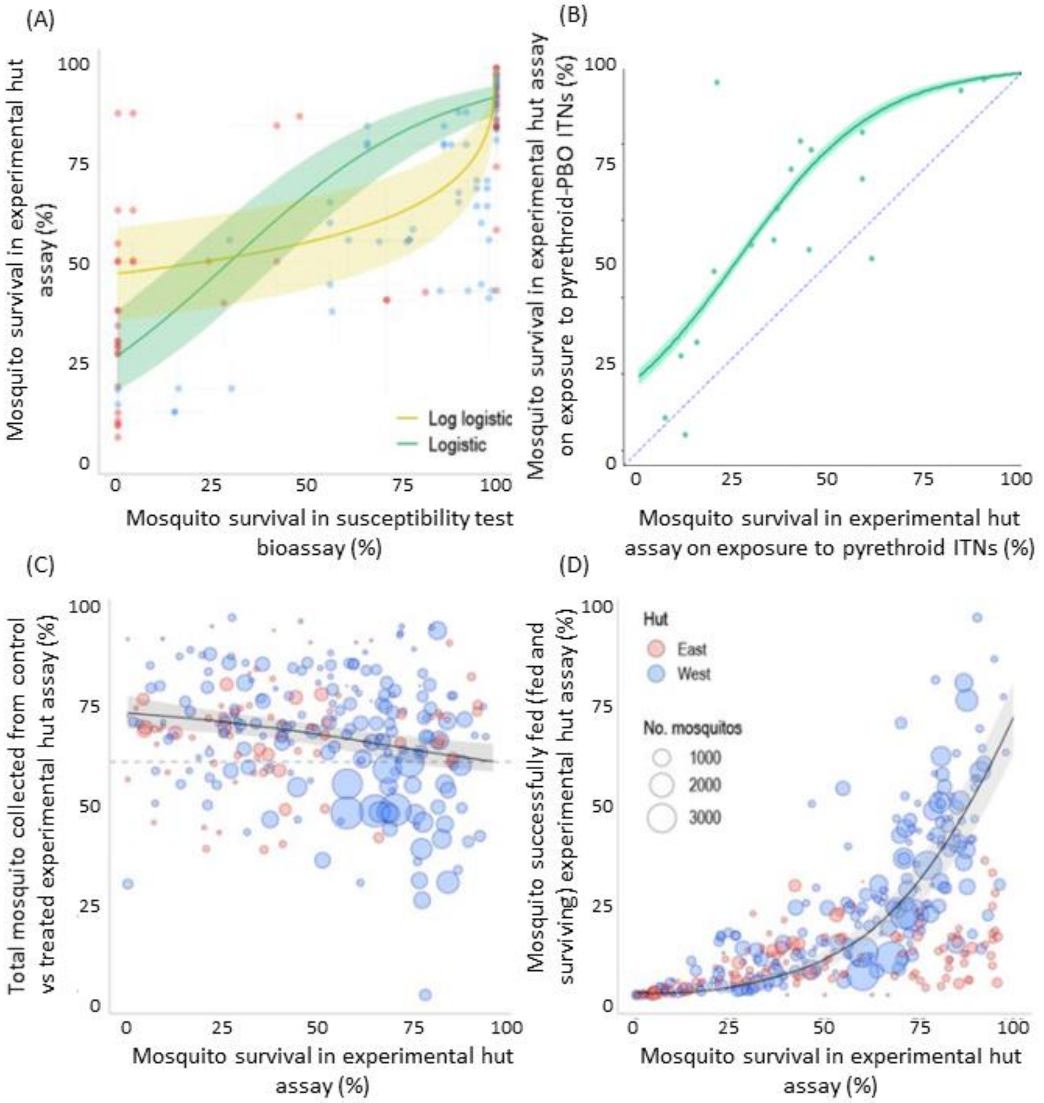

Statistical assessment of entomological data testing treated mosquito net products. (A) The association between mosquito mortality in a susceptibility bioassay and an experimental hut bioassay in the presence of pyrethroid nets. The variability in the data means there is no statistical reason to preferentially use either a logistic or log-logistic function to associate the data[1]. (B) The association between pyrethroid-PBO ITNs and pyrethroid-only nets indicating the advantage of the synergist when compared in experimental hut trials, EHTs (Eqn. S1.3). Mosquito survival in EHTs, regardless of hut design is associated with the ratio of mosquitoes caught in treated and control huts (B). This ratio is used to estimate how the probability of deterrence alters with induced mortality (Eqn. S1.4) which is itself related to both the type of net deployed and the level of resistance in the mosquito population (as approximated using susceptibility bioassays (A)). Similarly, the probability of mosquitoes successfully feeding (D) is associated with mosquito mortality in the EHTs (Eqn. S1.5). In panels C and D, colour reflects the design of EHT used (West African, blue; or East African, red). The best-fit (median and 90% Credible Intervals) shown represents the combined data weighted by number of mosquitoes recorded (size of points). Figures reproduced from Nash et al.[1].

Supplementary Figure S17.

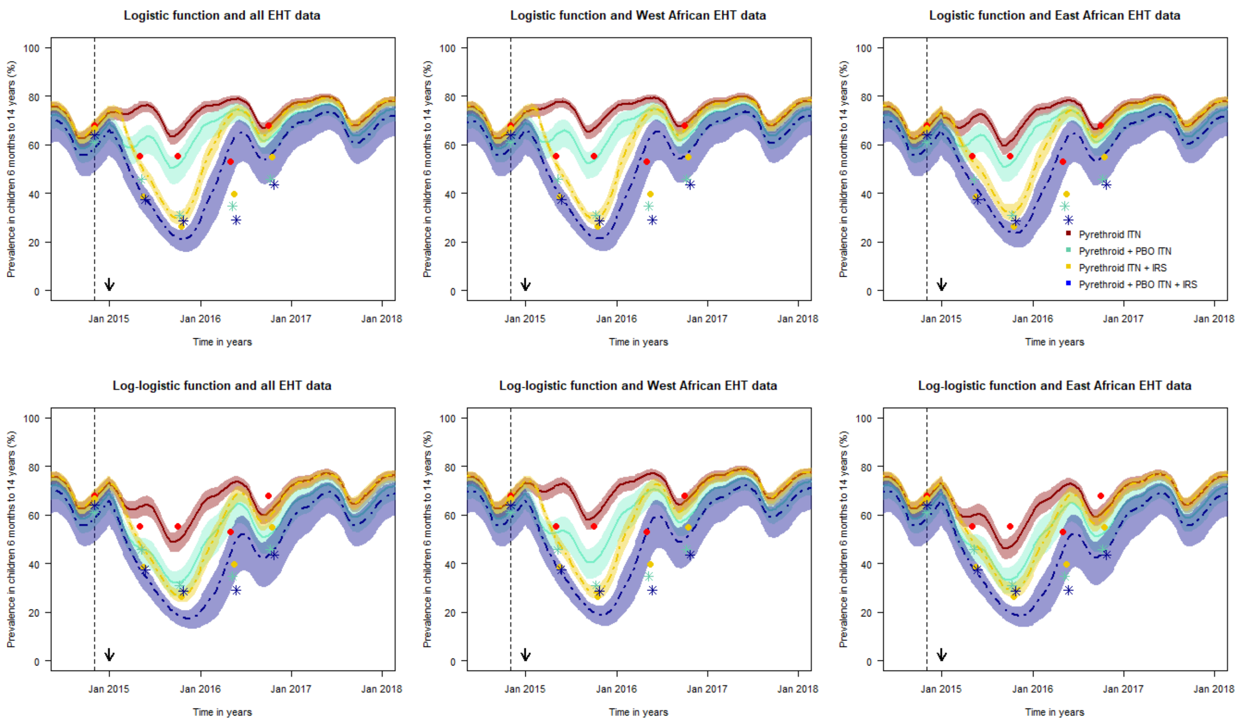

Sensitivity analysis of the different parameters derived from using either the logistic (top row) or log-logistic (bottom row) function to associate mortality during the susceptibility bioassay (as an approximate estimate of pyrethroid resistance) and mortality in the experimental hut trial EHT bioassay (as an estimate of the entomological efficacy of ITNs). The probable outcome of a mosquito feeding attempt is determined using EHT data. We fitted associations to predict these outcomes using all data (both West African design and East African design, first column), or West African design hut data only (middle column), or East African hut design only (final column) (see Materials and Methods). As an example, we show here the randomized control trial conducted in Muleba, Tanzania[23] because this study considered pyrethroid-nets (red circles), pyrethroid-PBO ITNs (green asterisks), pyrethroid-nets and one year of spraying of Actellic 300®CS IRS (yellow circles), and pyrethroid-PBO ITN and one year of spraying of Actellic 300®CS IRS (blue asterisks), thus representing the range of interventions we are considering across this systematic review and sensitivity analysis. The best performing analysis and resource were the log-logistic function and combined data (bottom left panel). The third cross sectional survey at 18 months post deployment (Jun 2016) is consistently missed (and can be seen in Supplementary Figure S18 as the asterisks, below the line of equivalence indicating the model underestimates efficacy at this time point), but the order of interventions is accurate and the 6 month, 12 month and 18 month surveys are reasonably captured with the framework.

Supplementary Figure S18

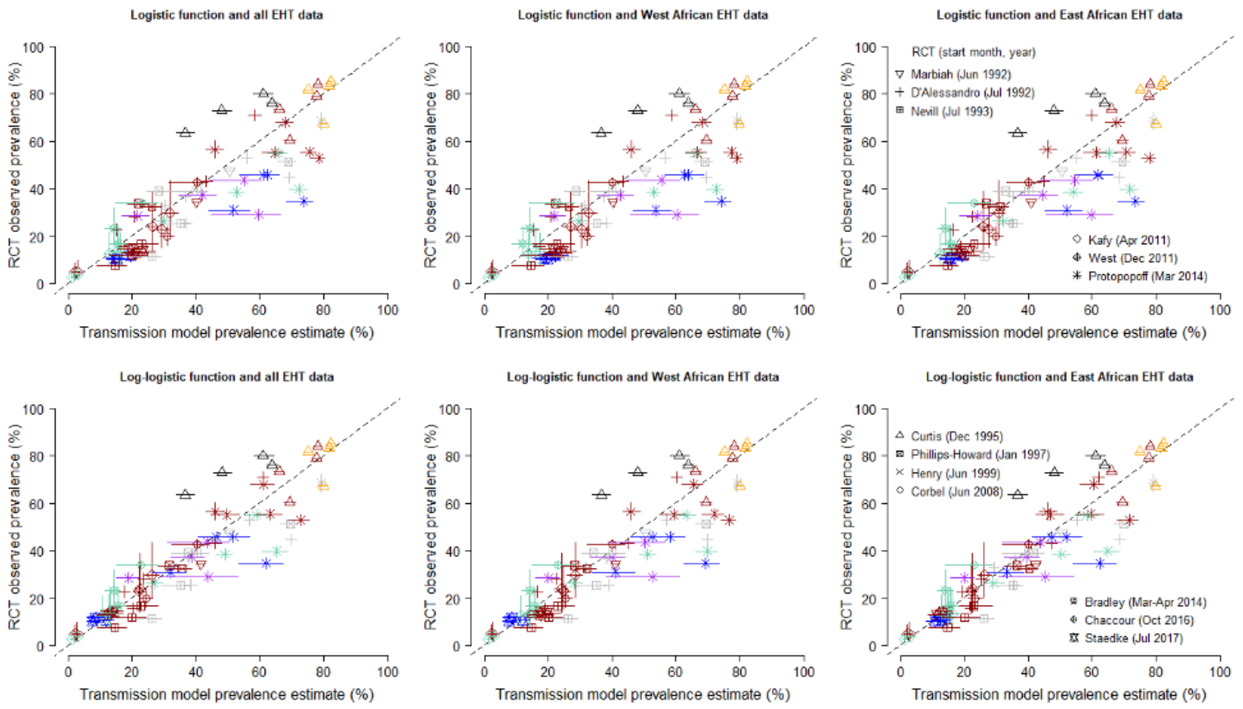

The predictive accuracy of the transmission model when parameterized using entomological correlates summarized by different functions and data resources (combined data – first column; West African hut data – central column; East African hut data – final column). Either the logistic growth (Eqn S1.1, top row of panels) or log-logistic (Eqn. 1.2, bottom row of panels) function is used to associate the mortality observed in susceptibility bioassay tests with mortality induced by the presence of a pyrethroid-only net in experimental hut bioassays. The corresponding associations between the entomological outcome of a mosquito feeding attempt in experimental huts with ITNs or IRS relative to those without any intervention determine the parameter estimates deployed in model simulations. Points represent observations made during randomized control trials (RCTs) testing different nets or IRS interventions. Model predicted uncertainty is driven by observed uncertainty from trial baseline data. Each RCT is represented by a distinct symbol as noted in Figure 1 and 2 of the main manuscript. Briefly, from top to bottom, circle[15], point-up triangle[16], cross[17], x[18], diamond[19], point-down triangle[20], square with x[22], asterisk[23], diamond with cross[25], circle with cross[26], star[24], square with cross[21], circle with x[13]. The colours denote the type of intervention examined; pyrethroid nets (conventionally treated dip nets or pyrethroid long-lasting treated nets; red), pyrethroid nets and IRS (green), pyrethroid-PBO ITNs (blue), pyrethroid PBO ITN and IRS (purple) or IRS only (orange). The association with intercept 0 is indicated by the adjusted-R^2, the closer to 1 the better the predictive performance of the model. Combining data from both West and East African huts and using a log-logistic function to associate mortality metrics provides the best predictive performance of the transmission model. Uncertainty estimates for the observed data are from the original publications found in Supplementary Data S1.3 and for the different models in Supplementary Data S1.7 for the different models.

Supplementary Figure S19

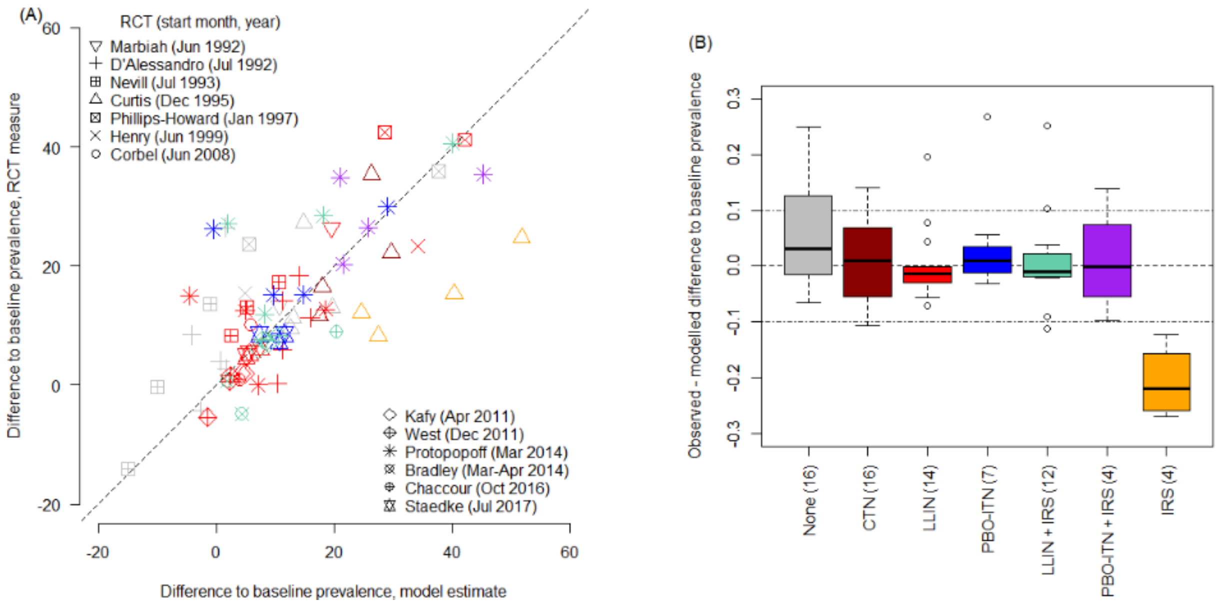

The accuracy of the model at predicting the absolute difference in malaria prevalence from baseline. The change in prevalence relative to the baseline observation may be a better way to assess the model performance at predicting the observed empirical data from the trials. A) The cluster-randomised trial (RCT) prevalence observed at cross-sectional surveys after deployment of the intervention and relative to baseline prevalence for each study (Eqn. S1.28) is plotted against the model simulated equivalent estimate. Symbols indicate the respective study (Supplementary Table 1), colours reflect specific interventions B) The absolute difference in the observed and modelled estimate for difference in prevalence relative to baseline prevalence for each type of intervention; none (16 data from 6 RCTs), conventionally treated dip-nets (16 data from 6 RCTs), long-lasting pyrethroid treated nets (14 data from 6 RCTs), pyrethroid-PBO ITNs (7 data from 2 RCTs), pyrethroid nets with additional IRS (12 data from 5 RCTs), pyrethroid-PBO ITNs with additional IRS (4 data from 1 RCT from 2014[23]) and IRS alone from a single RCT from 1995[5]. The box and whisker plots show the median (centre, black line), the interquartile range (difference between the 25th and 75th percentile, coloured box), reasonable range (1.5 times the interquartile range, whiskers) whilst the circles show observed minima and maxima outside of this range. This framework over-estimates the effect of IRS only from the study in 1995 whereas for all other single or combinations of interventions, the predictions is robust.

Supplementary Table 1.

Unique identifier	Principle Investigator (Study key reference, also see Data S1)	Location, Country (Start of trial or baseline data collection)	Trial arms, products tested	Malaria indicator + Age group
1	Bradley [13]	Bioko Island, Equatorial Guinea (March 2014)	1 Pyrethroid ITN + IRS: bendiocarb<br>2 Pyrethroid ITN + IRS: pyrethroid<br>Ficam 80 IRS (Bayer),<br>Deltamethrin IRS (Bayer)	Prevalence in 2 – 14-year olds
2	Chaccour [14]	Mopeia, Zambesia, Mozambique (Oct 2016)	1 Pyrethroid ITN (Control)<br>2 Pyrethroid ITN + IRS: organophosphate<br>ITN brand not specified. Deltamethrin-based ITNs (probably PermaNet 2.0, Vestergaard, Switzerland)<br>Actellic®300CS (Syngenta)	Prevalence in all ages; Clinical incidence, cases per 10,000 child-months (children under 5 years)
3	*Corbel* [15]	Ouidah-Kpomassè-Tori Health Area, Southern Benin, Benin (Jun 2008)	ⱡ1 Pyrethroid ITN-targeted to pregnant women and children under 6 years<br>2 Pyrethroid ITN-universal cover<br>ⱡ3 Pyrethroid ITN-targeted + IRS: carbamate<br>ⱡ4 Pyrethroid ITN-universal + carbamate treated plastic sheeting<br>PermaNet 2.0 ITNs (Vestergaard, Switzerland),<br>Ficam 80 IRS (Bayer),<br>Polypropylene CTPS (Filtisac SA, Cote D'Ivoire)	Prevalence *P. falciparum* under 6-years; Clinical incidence, cases per 100 child-months (children under 6 years)
4	Curtis [16]	Muheza, Tanga Region, Tanzania (Dec 1995 / Jan 1996)	1 No intervention (Control)<br>2 Dip-net ITNs, dipped every 8 months<br>3 IRS: Lambdacyhalothrin<br>100 denier knitted polyester (Siam Dutch Co., Holland)<br>Icon CS 10% IRS (Syngenta)	Prevalence (percentage positive for malaria parasites) 12-month to under 6-year olds
5	D'Alessandro [17]	The Gambia (Dec 1992)	1-5 Undipped bed nets (across 5 sites)<br>6-10 Dip-net ITN: permethrin (across 5 sites)	Parasite rates in children 1 – 4 years (baseline); Parasite rates in 'children' for post-intervention which may be 1-4 or 1-9 years
6	Henry [18]	Korhogo, Savanes, Côte D'Ivoire (Jun 1999)	1 Untreated bed nets<br>2 Dip-net ITN: lambda-cyhalothrin	Prevalence from blood smears in children under 5-years
7	Kafy [19]	Galabat, Sudan (Apr 2011)	1 Pyrethroid ITN<br>2 Pyrethroid ITN + IRS: deltamethrin, then bendiocarb<br>Permanet 2.0 ITNs (Vestergaard, Switzerland),<br>Deltamethrin 25 mg a.i. / m^2 (Chema Industries),	Clinical Incidence, cases per 1,000 child-years, RDT parasite prevalence in 6-month to 10-years.

			Ficam 80 IRS (Bayer, Germany)	
8‡	*Loha* [27]	Adami Tullu, East Shewa Zone, Oromia Region, Ethiopia (Jun 2014)	1 ᵻPyrethroid ITN + IRS<br>2 ᵻPyrethroid ITN<br>3 ᵻIRS<br>4 ᵻNo intervention (minimal ITN use)<br>Permanet 2.0 ITNs (Vestergaard, Switzerland),<br>ᵻPropoxur (Carbamate) IRS	All age clinical incidence, cases per 1,000-person years
9	Marbiah [20]	Bo district, Southern Province, Sierre Leone (Jun 1992)	1 No intervention<br>2 Pyrethroid ITN<br>Lambda-cyhalothrin $10mg/m^2$ (Siamdutch Mosquito Netting Co. Ltd, Bangkok, Thailand)	Prevalence in Under 7 year olds; Incidence per 1,000 child weeks at risk
10	Nevill [21]	Kilifi district, Kenya (Jun 1993)	1 No intervention<br>2 Permethrin-dip ITNs<br>Dip ITNs (SiamDutch, Bangkok, Thailand)	Mortality in children under 5 years; cases per 1,000 children per year
11	Phillips-Howard [28]	Asembo and Gem, Western Kenya (Jan 1997, Jan 1998)	1 Nothing, then permethrin-dip ITNs in year 2<br>2 Permethrin-dip ITNs<br>Staggered trial so that Asembo started Jan 1997 (arm 2), and Gem in Jan 1998 (arm 3).	Child-mortality; Parasite prevalence by microscopy in under 5-year olds
12	Protopopoff [23]	Muleba, Kagera, Tanzania (Mar 2014 (ITNs and IRS in Jan / Feb 2015))	1 Pyrethroid ITN<br>2 Pyrethroid plus PBO ITN<br>3 Pyrethroid ITN + IRS<br>4 Pyrethroid plus PBO ITN + IRS<br>Olyset Nets (Sumitomo Chemicals) with 2% w/w permethrin<br>Olyset Plus (Sumitomo Chemical) with 2% w/w permethrin and 1% PBO, Actellic 300CS IRS (Syngenta) with pirimiphos-methyl	Prevalence in 6-month to 14-year old children
13	Staedke [24]	Uganda	1 Deltamethrin ITNs (PermaNet 2.0) OR Permethin ITNs (Olyset®)<br>2 Deltamethrin + PBO ITNs (PermaNet 3.0) OR Permethin + PBO ITNs (Olyset®Plus)	Parasite prevalence (microscopy) in children aged 2–10 years
14	West [25]	Muleba, Kagera, Tanzania (Mar 2011)	1 Pyrethroid ITNs<br>2 Pyrethroid ITNs + IRS<br>Olyset nets (Sumitomo Chemicals) with 2% w/w permethrin ;<br>Lambda-cyhalothrin IRS (ICON 10CS, Syngenta) in 2011;<br>bendiocarb IRS (Ficam 80% wettable powder, Bayer) in 2012	Prevalence in children 6 months to 14 years (% *Pf*PR from Rapid diagnostic tests)

RDT – Rapid diagnostic test. ‡No prevalence measurement for the study. $Not included because DDT has not been characterised in the current modelling structure. ᵻNot included as the model is not structured to considered targeted distributions and has not been characterised for curtain intervention.

Cluster randomized control trials identified for model validation, and reasons for excluding studies where it was not possible to include them. Full details of the publications on each RCT identified in the systematic review are recorded in Data S1.1. Data S1.3 shows key data extracted to parameterize the model for simulations.

Supplementary Table 2.

Equation	Experimental hut data resource	Parameter	Value
S1.1	Combined East and West	β_{1a}<br>β_{2a}	3.57 (3.09 – 4.04)<br>0.70 (0.60 – 0.82)
S1.1	West	β_{1a}<br>β_{2a}	3.78 (3.16 – 4.39)<br>0.73 (0.62 – 0.84)
S1.1	East	β_{1a}<br>β_{2a}	3.28 (2.52 – 4.05)<br>0.65 (0.39 – 0.94)
S1.2	Combined East and West	β_{1b}<br>β_{2b}	0.77 (0.33 – 1.84)<br>0.47 (0.40 – 0.55)
S1.2	West	β_{1b}<br>β_{2b}	0.69 (0.37 – 1.21)<br>0.87 (0.71 – 1.06)
S1.2	East	β_{1b}<br>β_{2b}	0.64 (0.12 – 3.10)<br>0.35 (0.28 – 0.43)
S1.3	-	ω_1<br>ω_2	-1.82 (-1.88 – -1.76)<br>6.39 (6.16 – 6.61)
S1.4	Combined East and West	δ_1<br>δ_2<br>δ_3	2.44 (0.46 – 5.13)<br>0.42 (0.09 – 1.13)<br>0.36 (0.27 – 0.45)
S1.4	West	δ_1<br>δ_2<br>δ_3	3.05 (0.90 – 5.62)<br>0.57 (0.13 – 1.51)<br>0.39 (0.27 – 0.51)
S.14	East	δ_1<br>δ_2<br>δ_3	1.55 (0.13 – 4.21)<br>0.04 (0.00 – 0.26)<br>0.45 (0.37 – 0.53)
S1.5	Combined East and West	ρ_1<br>ρ_2	4.66 (4.51 – 4.81)<br>0.04 (0.03 – 0.05)
S1.5	West	ρ_1<br>ρ_2	5.13 (4.96 – 5.30)<br>0.04 (0.03 – 0.04)
S1.5	East	ρ_1<br>ρ_2	3.43 (3.12 – 3.76)<br>0.01 (0.00 – 0.02)
S1.6 – S1.8	-	k_0	0.699[3]
S1.9	-	$\mu_{p=2,\ pyrethroid\text{-}PBO\ ITN}$<br>$\rho_{p=2,\ pyrethroid\text{-}PBO\ ITN}$	-2.43 (-2.98 – -1.87)[2]<br>-3.01 (-3.74 – -2.30)

Model median parameter estimates from the statistical analysis characterising the diminishing protection provided by ITNs when mosquitoes are resistant to pyrethroid insecticides. Resistance is assumed to be approximate to the proportion of mosquitoes surviving exposure to a discriminatory dose of pyrethroid in a susceptibility bioassay. This association is reasonably described by a logistic (Eqn. S1.1) or log-logistic (Eqn. S1.2) function. Pyrethroid-ITN mortality at experimental hut assessment is associated with pyrethroid-PBO ITN induced mortality (Eqn. S1.3). The entomological outcome of a mosquito feeding attempt is determined by the associations between mortality induced in the experimental hut and successful blood-feeding (Eqn. S1.5) or deterrence of the mosquito (Eqn. S1.4). Eqns. 1.6-1.8 combine these estimates using parameter k_0 which is assumed to represent the probability of successful feeding in the absence of any intervention.[3] The duration of this impact is determined in Eqn. S1.9 specific for each impact.

Supplementary Table 3

	Method, logistic			Method, log-logistic		
	All EHTs	West African EHTs	East African EHTs	All EHTs	West African EHTs	East African EHTs
	Can the model predicted prevalence (X) predict the observed RCT data (Y): $Y_i = mX_i + 0$, where *i* are individual repeated measures for each trial (Eqn. S1.26)					
All interventions (n = 73)	m = 0.90<br>Adj-R^2 = 0.93	m = 0.89<br>Adj-R^2 = 0.93	m = 0.91<br>Adj-R^2 = 0.94	**m = 0.96**<br>**Adj-R^2 = 0.95**	m = 0.92<br>Adj-R^2 = 0.94	**m = 0.96**<br>**Adj-R^2 = 0.95**
Any ITN, no IRS (n = 37)	m = 0.88<br>Adj-R^2 = 0.94	m = 0.86<br>Adj-R^2 = 0.93	m = 0.88<br>Adj-R^2 = 0.95	**m = 0.97**<br>**Adj-R^2 = 0.97**	m = 0.92<br>Adj-R^2 = 0.96	m = 0.96<br>**Adj-R^2 = 0.97**
Long-lasting pyrethroid nets (n = 14)	m = 0.81<br>Adj=R^2 = 0.97	m = 0.79<br>Adj-R^2 = 0.97	m = 0.84<br>Adj-R^2 = 0.97	m = 0.95<br>**Adj-R^2 = 0.97**	m = 0.86<br>Adj-R^2 = 0.97	**m = 0.97**<br>**Adj-R^2 = 0.97**
Pyrethroid-PBO nets (n = 7)	m = 0.63<br>Adj-R^2 = 0.96	m = 0.61<br>Adj-R^2 = 0.96	m = 0.63<br>Adj-R^2 = 0.96	**m = 0.80**<br>Adj-R^2 = 0.93	m = 0.70<br>**Adj-R^2 = 0.94**	m = 0.78<br>**Adj-R^2 = 0.94**
Any IRS (n = 20)	m = 0.95<br>Adj-R^2 = 0.87	m = 0.94<br>Adj-R^2 = 0.86	m = 0.94<br>Adj-R^2 = 0.87	m = 1.06<br>**Adj-R^2 = 0.91**	**m = 0.99**<br>Adj-R^2 = 0.87	m = 1.04<br>**Adj-R^2 = 0.91**
	Can the model predicted efficacy against prevalence (E_j^X) predict the observed RCT data (E_j^Y): $E_j^Y = mE_j^X + 0$, where *j* are individual repeated measures for each trial (Eqn. S1.27)					
All interventions (n = 46)	m = 0.73<br>Adj-R^2 = 0.63	m = 0.59<br>Adj-R^2 = 0.67	m = 0.78<br>Adj-R^2 = 0.61	**m = 0.78**<br>**Adj-R^2 = 0.67**	m = 0.71<br>Adj-R^2 = 0.63	**m = 0.85**<br>**Adj-R^2 = 0.63**
Any ITN, no IRS (n = 23)‡	m = 0.81<br>Adj-R^2 = 0.58	m = 0.81<br>Adj-R^2 = 0.57	m = 0.91<br>Adj-R^2 = 0.58	**m = 0.84**<br>**Adj-R^2 = 0.64**	m = 0.71<br>Adj-R^2 = 0.56	**m = 1.00**<br>**Adj-R^2 = 0.62**
Pyrethroid-PBO nets (n = 7)	m = 1.25<br>Adj-R^2 = 0.63	m = 1.87<br>Adj-R^2 = 0.76	m = 2.24<br>Adj-R^2 = 0.78	**m = 0.88**<br>**Adj-R^2 = 0.68**	m = 0.57<br>Adj-R^2 = 0.45	m = 1.55<br>Adj-R^2 = 0.67
Any IRS (n = 23)	m = 0.69<br>Adj-R^2 = 0.66	m = 0.60<br>Adj-R^2 = 0.62	m = 0.71<br>Adj-R^2 = 0.65	m = 0.73<br>**Adj-R^2 = 0.70**	m = 0.70<br>Adj-R^2 = 0.68	**m = 0.77**<br>Adj-R^2 = 0.64

* These include conventionally treated dipped nets and long-lasting pyrethroid nets. ‡All nets were conventionally treated dipped nets or pyrethroid-PBO ITNs (none were long-lasting pyrethroid treated nets).

Results of the sensitivity analysis to predict the observed prevalence or efficacy against prevalence over time measured in cluster-randomized control trials (RCTs). Given the uncertainty in the entomological data collated from different resources, we explored the combination of entomological data and statistical associations that produce parameters with the greatest predictive power. There are 6 combinations of analysis and data; columns 1 to 3 combine entomological data for mosquito mortality in susceptibility tests and experimental hut assays after exposure to pyrethroid discriminatory doses or pyrethroid ITNs using a logistic function. Columns 4 to 6 apply a log-logistic function to associate these data (Materials and Methods). Experimental hut trials can be performed in West or East African style huts, these data have been systematically reviewed in Nash et al [1]. Here, we use associations fitted to all these data (columns 1 and 4), only West African (columns 2 and 5), or only East African (columns 3 and 6) to determine parameter estimates for the transmission model (Supplementary Data). After simulating the respective trial arms (Figures S4-S16), the mean observed estimates for the respective age-cohort and cross-sectional surveys conducted in the trials are associated with the corresponding model predicted mean estimates (Figure 2B) using linear regression. In each analysis, the closer the gradient m and the adjusted-R^2 (adj=R^2) are to unity the more accurate the predictive performance of the mechanistic model parameterisation (Methods). Best-performing parameter sets are highlighted.

Supplementary Table 4.

West African RCTs (N = 17 data points)			East African RCTs (N = 52 data points)		
All EHT data	West Afr. EHT	East Afr. EHT	All EHT data	West Afr. EHT	East Afr. EHT
Can the model predicted prevalence (X) predict the observed RCT data (Y): $Y_i = mX_i + 0$, where *i* are individual repeated measures for each trial (Equation 2)					
m = 0.92<br>**Adj-R² = 0.96**	*m* = 0.92<br>**Adj-R² = 0.96**	*m* = 0.93<br>**Adj-R² = 0.96**	*m* = 0.97<br>**Adj-R² = 0.95**	*m* = 0.93<br>Adj-R² = 0.94	*m* = 0.97<br>**Adj-R² = 0.95**
Can the model predicted efficacy against prevalence (E_j^X) predict the observed RCT data (E_j^Y): $E_j^Y = mE_j^X + 0$, where *j* are individual repeated measures for each trial (Eqn. 3)					
West African RCTs (N = 8 data points)			East African RCTs (N = 36 data points)		
m = 0.55<br>**Adj-R² = 0.19**	*m* = 0.52<br>**Adj-R² = 0.19**	*m* = 0.63<br>Adj-R² = 0.17	*m* = 0.81<br>**Adj-R² = 0.79**	*m* = 0.73<br>Adj-R² = 0.73	*m* = 0.89<br>Adj-R² = 0.72

Results of the sensitivity analysis using parameters sets determined from different statistical analyses of entomological data, or different data resource combinations, to predict the observed prevalence or efficacy against prevalence over time measured in randomized control trials (RCTs) performed in either West or East African countries. The log-logistic function (the best-performing function) and the combined data, West African design experimental hut (EHT) data, or East African design EHT data. In each linear regression, the closer adjusted-R^2 (adj-R^2) is to unity the more accurate the predictive performance of the mechanistic model (highlighted in bold). We find no evidence to indicate that local hut design improves model predictions. Care should be taken interpreting these results particularly as so few RCT data points are in West Africa. The previous systematic review identified more EHT studies conducted using the West African design [1] though the current systematic review had more data from RCTs from the East Africa region that fulfilled the search criteria.

References

1. Nash, R. K. *et al.* Systematic review of the entomological impact of insecticide-treated nets evaluated using experimental hut trials in Africa. *Current Research in Parasitology & Vector-Borne Diseases* **1**, 100047 (2021).
2. Churcher, T. S., Lissenden, N., Griffin, J. T., Worrall, E. & Ranson, H. The impact of pyrethroid resistance on the efficacy and effectiveness of bednets for malaria control in Africa. *eLife* **5**, (2016).
3. Griffin, J. T. *et al.* Reducing Plasmodium falciparum malaria transmission in Africa: a model-based evaluation of intervention strategies. *PLoS medicine* **7**, e1000324 (2010).
4. Lines, J. D., Myamba, J. & Curtis, C. F. Experimental hut trials of permethrin-impregnated mosquito nets and eave curtains against malaria vectors in Tanzania. *Medical and Veterinary Entomology* **1**, 37–51 (1987).
5. Curtis, C. F., Myamba, J. & Wilkes, T. J. Comparison of different insecticides and fabrics for anti-mosquito bednets and curtains. *Medical and Veterinary Entomology* **10**, 1–11 (1996).
6. Mathenge, E. M. *et al.* Effect of Permethrin-Impregnated Nets on Exiting Behavior, Blood Feeding Success, and Time of Feeding of Malaria Mosquitoes (Diptera: Culicidae) in Western Kenya. *Journal of Medical Entomology* **38**, 531–536 (2001).
7. Mahama, T., Desiree, E. J., Pierre, C. & Fabrice, C. Effectiveness of permanet in Côte d'Ivoire rural areas and residual activity on a knockdown-resistant strain of Anopheles gambiae. *Journal of medical entomology* **44**, 498–502 (2007).
8. Sherrard-Smith, E. *et al.* Systematic review of indoor residual spray efficacy and effectiveness against Plasmodium falciparum in Africa. *Nature Communications* **9**, 4982 (2018).
9. Sherrard-Smith, E. *et al.* The potential public health consequences of COVID-19 on malaria in Africa. *Nature Medicine* 1–6 (2020) doi:10.1038/s41591-020-1025-y.
10. Bagi, J. *et al.* When a discriminating dose assay is not enough: measuring the intensity of insecticide resistance in malaria vectors. *Malaria Journal* **14**, 210 (2015).
11. Massue, D. J. *et al.* Comparative performance of three experimental hut designs for measuring malaria vector responses to insecticides in Tanzania. *Malaria journal* **15**, 165 (2016).
12. World Health Organization. Malaria Threats Map. https://apps.who.int/malaria/maps/threats (2021).
13. Bradley, J. *et al.* A cluster randomized trial comparing deltamethrin and bendiocarb as insecticides for indoor residual spraying to control malaria on Bioko Island, Equatorial Guinea. *Malaria Journal* **15**, 378 (2016).
14. Chaccour, C. *et al.* Incremental impact on malaria incidence following indoor residual spraying in a highly endemic area with high standard ITN access in Mozambique: results from a cluster-randomized study. *Malaria Journal* **20**, 1–15 (2021).
15. Corbel, V. *et al.* Combination of malaria vector control interventions in pyrethroid resistance area in Benin: a cluster randomised controlled trial. *The Lancet Infectious Diseases* **12**, 617–626 (2012).
16. Curtis, C. F. *et al.* A comparison of use of a pyrethroid either for house spraying or for bednet treatment against malaria vectors. *Tropical Medicine & International Health* **3**, 619–631 (1998).
17. D'Alessandro, U. *et al.* Mortality and morbidity from malaria in Gambian children after introduction of an impregnated bednet programme. *The Lancet* **345**, 479–483 (1995).
18. Henry, M. C. *et al.* Protective efficacy of lambda-cyhalothrin treated nets in Anopheles gambiae pyrethroid resistance areas of Côte d'Ivoire. *American Journal of Tropical Medicine and Hygiene* **73**, 859–864 (2005).
19. Kafy, H. T. *et al.* Impact of insecticide resistance in Anopheles arabiensis on malaria incidence and prevalence in Sudan and the costs of mitigation. *Proceedings of the National Academy of Sciences of the United States of America* **114**, E11267–E11275 (2017).
20. Marbiah, N. T. *et al.* A controlled trial of lambda-cyhalothrin-impregnated mosquito bed nets and/or dapsone/pyrimethamine for malaria control in Sierra Leone. *American Journal of Tropical Medicine and Hygiene* **58**, 1–6 (1998).
21. Nevill, C. G. *et al.* Insecticide-treated bednets reduce mortality and severe morbidity from malaria among children on the Kenyan coast. *Tropical Medicine & International Health* **1**, 139–146 (1996).
22. Phillips-Howard, P. A. *et al.* The efficacy of permethrin-treated bed nets on child mortality and morbidity in western Kenya I. Development of infrastructure and description of study site. *American Journal of Tropical Medicine and Hygiene* **68**, 3–9 (2003).
23. Protopopoff, N. *et al.* Effectiveness of a long-lasting piperonyl butoxide-treated insecticidal net and indoor residual spray interventions, separately and together, against malaria transmitted by pyrethroid-resistant mosquitoes: a cluster, randomised controlled, two-by-two fact. *The Lancet* **391**, 1577–1588 (2018).

24. Staedke, S. G. *et al.* Effect of long-lasting insecticidal nets with and without piperonyl butoxide on malaria indicators in Uganda (LLINEUP): a pragmatic, cluster-randomised trial embedded in a national LLIN distribution campaign. *The Lancet* **395**, 1292–1303 (2020).
25. West, P. A. *et al.* Indoor Residual Spraying in Combination with Insecticide-Treated Nets Compared to Insecticide-Treated Nets Alone for Protection against Malaria: A Cluster Randomised Trial in Tanzania. *PLoS Medicine* **11**, e1001630 (2014).
26. Chaccour, C. J. *et al.* Combination of indoor residual spraying with long-lasting insecticide-treated nets for malaria control in Zambezia, Mozambique: A cluster randomised trial and cost-effectiveness study protocol. *BMJ Global Health* **3**, (2018).
27. Loha, E. *et al.* Long-lasting insecticidal nets and indoor residual spraying may not be sufficient to eliminate malaria in a low malaria incidence area: Results from a cluster randomized controlled trial in Ethiopia. *Malaria Journal* **18**, 1–15 (2019).
28. Phillips-Howard, P. A. *et al.* The efficacy of permethrin-treated bed nets on child mortality and morbidity in western Kenya I. Development of infrastructure and description of study site. *American Journal of Tropical Medicine and Hygiene* **68**, 3–9 (2003).
29. Sherrard-Smith, E. *et al.* Optimising the deployment of vector control tools against malaria: a data-informed modelling study. *The Lancet Planetary Health* (2022) doi:10.1016/S2542-5196(21)00296-5.
30. Winskill, P., Walker, P. G., Griffin, J. T. & Ghani, A. C. Modelling the cost-effectiveness of introducing the RTS,S malaria vaccine relative to scaling up other malaria interventions in sub-Saharan Africa. *BMJ Global Health* **2**, (2017).
31. Walker, P. G. T., Griffin, J. T., Ferguson, N. M. & Ghani, A. C. Estimating the most efficient allocation of interventions to achieve reductions in Plasmodium falciparum malaria burden and transmission in Africa: a modelling study. *The Lancet Global Health* **4**, e474-84 (2016).
