# Supplementary material for: Inferring the epidemiological benefit of indoor vector control interventions against malaria from mosquito data
Source: Nat Commun. 2022 Jul 5;13:3862. doi: 10.1038/s41467-022-30700-1 (PMC9256631; doi:10.1038/s41467-022-30700-1)
Supplement: Supplementary file 2 — Description of Additional Supplementary Files [file 41467_2022_30700_MOESM2_ESM.pdf]

Inferring the epidemiological benefit of indoor vector control interventions against malaria from mosquito data

Authors: E Sherrard-Smith[1], Corine Ngufor[2,3], Antoine Sanou[4], Moussa Guelbeogo[4], Raphael N'Guessan[5,3], Eldo Elobolobo[6], Francisco Saute[6], Kenyssony Varela[7], Carlos Chaccour[8], Rose Zulliger[9], Joe Wagman[10], Molly L. Robertson[10], Mark Rowland[3], Martin Donnelly[11], Samuel Gonahasa[12], Sarah Staedke[3], Jan Kolaczinski[13], Thomas S. Churcher*[1].

Supplementary Data S1 Legend.

S1.1 Systematic Review Papers

Summary of the 138 resources identified for further assessment in the systematic review. A list of the key publications and reasons for exclusion.

S1.2 Study data resource

A total of 14 randomized control trials were identified that were suitable to include in the assessment of model capacity to predict randomized control trial epidemiological results from entomological data. Here, we flag which of the key data are available from each RCT and the associated resource providing these data. Thirteen had prevalence cross-sectional surveys.

S1.3 Study data

Average and range of data recorded in each of the 13 randomized control trials (one trial does not report prevalence) that are used in the model validation process

S1.4 Efficacy parameters ITNs

Parameter estimates that are determined using a systematic review of experimental hut data testing the entomological impact of insecticide treated bed nets. These parameters, derived from the original review [1], show the probability of mosquitoes repeating after an unsuccessful feeding attempt, the probability of mosquitoes being killed in the presence of the ITN and the half-life of the active ingredient on the ITNs for increasing pyrethroid resistance (approximated by the proportion of mosquitoes surviving exposure to a discriminatory dose of pyrethroid during a 24-hour bioassay test) and for both pyrethroid-only, and pyrethroid plus PBO ITNs as defined in [29]. A sensitivity analysis was undertaken to determine the entomological correlates and analysis that provides the parameter set that can give most predictive accuracy for the model simulations. This is set 4 in the xlsx file.

S1.5 Efficacy parameters IRS

Parameter estimates that are determined using a systematic review of experimental hut data testing the entomological impact of insecticides sprayed indoors (IRS) [8]. These parameters show 1000 posterior draws from the model fitting process to capture the 80% credible intervals around the

mean for Actellic®300CS and Ficam®. The impact of pyrethroid IRS products are diminished in the presence of pyrethroid resistance. The adjustments made to parameter estimates with increasing resistance are shown for pyrethroid IRS. These parameters determine the shape of the relationship between induced mosquito mortality ($l_{s\vartheta}$, $l_{s\gamma}$), changes in successful blood-feeding ($k_{s\vartheta}$, $k_{s\gamma}$), and deterrence from houses ($m_{s\vartheta}$, $m_{s\gamma}$) with time since spraying the residual insecticide.

S1.6 RCT site parameters

A list of parameters specifying the baseline conditions for each randomized control trial arm that is simulated by the transmission model (for details of the model and default parameter estimates, see supplements for [3,30,31]).

S1.7 Summary data for observed and predicted prevalence

A summary of the mean, minimum and maximum trial observed, and model estimated, measures of parasite prevalence throughout each randomized control trial (RCT). Data are characterized by the time when observations were made, by the type of intervention explored and the location of study.

S1.8 Summary data for observed and predicted efficacy against prevalence

A summary of the mean, minimum and maximum trial observed and model estimated measures of efficacy against prevalence, matched for age-cohorts observed in bespoke trials, throughout each randomized control trial (RCT). Data are characterized by the time when observations were made, by the type of intervention explored and the location of study.
