# Supplementary material for: Inferring the epidemiological benefit of indoor vector control interventions against malaria from mosquito data
Source: Nat Commun. 2022 Jul 5;13:3862. doi: 10.1038/s41467-022-30700-1 (PMC9256631; doi:10.1038/s41467-022-30700-1)
Supplement: Supplementary file 5 — Reporting Summary [file 41467_2022_30700_MOESM5_ESM.pdf]

nature portfolio

Corresponding author(s): Tom Churcher
Last updated by author(s): Dec 16, 2021

Reporting Summary

Nature Portfolio wishes to improve the reproducibility of the work that we publish. This form provides structure for consistency and transparency in reporting. For further information on Nature Portfolio policies, see our Editorial Policies and the Editorial Policy Checklist.

Statistics

For all statistical analyses, confirm that the following items are present in the figure legend, table legend, main text, or Methods section.

n/a	Confirmed	
☐	☒	The exact sample size (n) for each experimental group/condition, given as a discrete number and unit of measurement
☒	☐	A statement on whether measurements were taken from distinct samples or whether the same sample was measured repeatedly
☒	☐	The statistical test(s) used AND whether they are one- or two-sided<br>*Only common tests should be described solely by name; describe more complex techniques in the Methods section.*
☐	☒	A description of all covariates tested
☐	☒	A description of any assumptions or corrections, such as tests of normality and adjustment for multiple comparisons
☐	☒	A full description of the statistical parameters including central tendency (e.g. means) or other basic estimates (e.g. regression coefficient) AND variation (e.g. standard deviation) or associated estimates of uncertainty (e.g. confidence intervals)
☒	☐	For null hypothesis testing, the test statistic (e.g. F, t, r) with confidence intervals, effect sizes, degrees of freedom and P value noted<br>*Give P values as exact values whenever suitable.*
☐	☒	For Bayesian analysis, information on the choice of priors and Markov chain Monte Carlo settings
☒	☐	For hierarchical and complex designs, identification of the appropriate level for tests and full reporting of outcomes
☐	☒	Estimates of effect sizes (e.g. Cohen's d, Pearson's r), indicating how they were calculated

Our web collection on statistics for biologists contains articles on many of the points above.

Software and code

Policy information about availability of computer code

Data collection	No model code was used for data extraction. Code outlining data manipulation is available here: https://github.com/EllieSherrardSmith/ibm_rct_prediction.
Data analysis	The transmission dynamics mathematical model is written in C++ and model code can be found here: https://github.com/jamiegriffin/Malaria_simulation and input parameters and processing code is available here: https://github.com/EllieSherrardSmith/ibm_rct_prediction.

For manuscripts utilizing custom algorithms or software that are central to the research but not yet described in published literature, software must be made available to editors and reviewers. We strongly encourage code deposition in a community repository (e.g. GitHub). See the Nature Portfolio guidelines for submitting code & software for further information.

Data

Policy information about availability of data

All manuscripts must include a data availability statement. This statement should provide the following information, where applicable:

- Accession codes, unique identifiers, or web links for publicly available datasets
- A description of any restrictions on data availability
- For clinical datasets or third party data, please ensure that the statement adheres to our policy

All data collated and generated during this study are included in this published article (Supplementary_Data_1).

Field-specific reporting

Please select the one below that is the best fit for your research. If you are not sure, read the appropriate sections before making your selection.

☒ Life sciences ☐ Behavioural & social sciences ☐ Ecological, evolutionary & environmental sciences

For a reference copy of the document with all sections, see nature.com/documents/nr-reporting-summary-flat.pdf

Life sciences study design

All studies must disclose on these points even when the disclosure is negative.

Sample size	The manuscript does not report primary data collection. No sample size calculations were conducted for the secondary data analyses as all studies identified through the systematic review were utilized. Given over 70 trial arms were identified it was felt this was sufficient to differentiate between the different models.
Data exclusions	The systematic review identified studies which were excluded from the main analysis. The reasons for this exclusion are provided in the methods section and Supporting Data S1.1 (where a reason for the exclusion from each arm is provided in column A. Exclusions are graphically represented in Supplementary Figure 1.
Replication	Individual-based simulation models were repeated 100,000 times and the median result presented. Ninety five percent confidence interval estimates are provided throughout and were sufficiently tight to justify the number of replications chosen and conclusions drawn.
Randomization	There was no randomization in this analysis consistent with the guidelines for meta-analyses (Cochrane Handbook, https://training.cochrane.org/handbook/)
Blinding	There was no blinding in this secondary analysis consistent with the methodology for meta-analyses (Cochrane Handbook, https://training.cochrane.org/handbook/).

Reporting for specific materials, systems and methods

We require information from authors about some types of materials, experimental systems and methods used in many studies. Here, indicate whether each material, system or method listed is relevant to your study. If you are not sure if a list item applies to your research, read the appropriate section before selecting a response.

Materials & experimental systems

n/a	Involved in the study	
☒	☐	Antibodies
☒	☐	Eukaryotic cell lines
☒	☐	Palaeontology and archaeology
☒	☐	Animals and other organisms
☒	☐	Human research participants
☒	☐	Clinical data
☒	☐	Dual use research of concern

Methods

n/a	Involved in the study	
☒	☐	ChIP-seq
☒	☐	Flow cytometry
☒	☐	MRI-based neuroimaging
