# Supplementary material for: Inferring the epidemiological benefit of indoor vector control interventions against malaria from mosquito data
Source: Nat Commun. 2022 Jul 5;13:3862. doi: 10.1038/s41467-022-30700-1 (PMC9256631; doi:10.1038/s41467-022-30700-1)
Supplement: Supplementary file 4 — Peer Review File [file 41467_2022_30700_MOESM4_ESM.pdf]

nature portfolio

Peer Review File

Inferring the epidemiological benefit of indoor vector control interventions against malaria from mosquito data

Open Access This file is licensed under a Creative Commons Attribution 4.0 International License, which permits use, sharing, adaptation, distribution and reproduction in any medium or format, as long as you give appropriate credit to the original author(s) and the source, provide a link to the Creative Commons license, and indicate if changes were made. In the cases where the authors are anonymous, such as is the case for the reports of anonymous peer reviewers, author attribution should be to 'Anonymous Referee' followed by a clear attribution to the source work. The images or other third party material in this file are included in the article's Creative Commons license, unless indicated otherwise in a credit line to the material. If material is not included in the article's Creative Commons license and your intended use is not permitted by statutory regulation or exceeds the permitted use, you will need to obtain permission directly from the copyright holder. To view a copy of this license, visit http://creativecommons.org/licenses/by/4.0/.

Editorial Note: This manuscript has been previously reviewed at another journal that is not operating a transparent peer review scheme. This document only contains reviewer comments and rebuttal letters for versions considered at *Nature Communications.*

REVIEWERS' COMMENTS

Reviewer #1 (Remarks to the Author):

Thank you for comprehensively addressing all my comments on a previous version of this manuscript.
I have no further comments to add, and recommend acceptance.

Reviewer #2 (Remarks to the Author):

This articles investigated whether data from experimental hut trials (EHTs) are comparable to data from cluster randomized trials (CRT) when evaluating new insecticide treated nets or insecticides. Based on their findings, the authors suggests that EHTs could be a cost-effective alternative to CRTs, particularly when assess new classes of ITNs. The research is novel, appears to be sound and, if accepted by the global malaria community could positively impact elimination efforts. I have no further comments at this stage.
